# MEIOB Targets Single-Strand DNA and Is Necessary for Meiotic Recombination

**DOI:** 10.1371/journal.pgen.1003784

**Published:** 2013-09-19

**Authors:** Benoit Souquet, Emilie Abby, Roxane Hervé, Friederike Finsterbusch, Sophie Tourpin, Ronan Le Bouffant, Clotilde Duquenne, Sébastien Messiaen, Emmanuelle Martini, Jacqueline Bernardino-Sgherri, Attila Toth, René Habert, Gabriel Livera

**Affiliations:** 1 Univ. Paris Diderot, Sorbonne Paris Cité, Laboratory of Development of the Gonads, Unit of Stem Cells and Radiation, UMR 967, Fontenay aux Roses, France; 2 CEA, DSV, iRCM, SCSR, LDG, Fontenay aux Roses, France; 3 INSERM, Unité 967, Fontenay aux Roses, France; 4 Univ. Paris-Sud, UMR 967, Fontenay aux Roses, France; 5 Molecular Cell Biology Group/Experimental Center, Institute of Physiological Chemistry, Medical School, MTZ, Dresden University of Technology, Dresden, Germany; INRA, France

## Abstract

Meiotic recombination is a mandatory process for sexual reproduction. We identified a protein specifically implicated in meiotic homologous recombination that we named: meiosis specific with OB domain (MEIOB). This protein is conserved among metazoan species and contains single-strand DNA binding sites similar to those of RPA1. Our studies *in vitro* revealed that both recombinant and endogenous MEIOB can be retained on single-strand DNA. Those *in vivo* demonstrated the specific expression of *Meiob* in early meiotic germ cells and the co-localization of MEIOB protein with RPA on chromosome axes. MEIOB localization in *Dmc1* ^−/−^ spermatocytes indicated that it accumulates on resected DNA. Homologous *Meiob* deletion in mice caused infertility in both sexes, due to a meiotic arrest at a zygotene/pachytene-like stage. DNA double strand break repair and homologous chromosome synapsis were impaired in *Meiob* ^−/−^ meiocytes. Interestingly MEIOB appeared to be dispensable for the initial loading of recombinases but was required to maintain a proper number of RAD51 and DMC1 foci beyond the zygotene stage. In light of these findings, we propose that RPA and this new single-strand DNA binding protein MEIOB, are essential to ensure the proper stabilization of recombinases which is required for successful homology search and meiotic recombination.

## Author Summary

Homologous recombination allows faithful repair of damaged DNA; in mitotic cells, it necessitates the formation of single strand DNA (ssDNA), which is first protected by RPA and then coated by the RAD51 recombinase to mediate homology search. Specific modifications are made to this mechanism during meiosis, a specialized division that allows halving the ploidy of the genome and the production of haploid gametes. Among others a specialized recombinase DMC1 is added to its somatic paralog RAD51 to perform homology search. We identified a new meiotic protein that we named meiosis specific with OB domains (MEIOB). Our findings indicate that MEIOB binds ssDNA, and we propose that MEIOB is a meiotic paralog of RPA, another OB-domain containing protein. *Meiob* mutant mice were infertile and unable to complete meiotic recombination, most likely due to destabilization of DMC1 and RAD51 in the absence of MEIOB. Meiosis appears thus to be a ‘game of two pairs’ using both the canonical players in homologous recombination (RPA and RAD51) and a second set of paralogs (MEIOB and DMC1). Identifying such new players should help clarify some genetic causes of infertility and shed new light on the interplay between the molecular actors involved in maintaining genome stability.

## Introduction

Meiosis is a central process of sexual reproduction. This specialized cell division program allows halving the genome of diploid germ cells to produce haploid gametes. In order to ensure proper segregation of homologous chromosomes during the first meiotic division, these must become connected through chiasmata [1]. Crucially, formation of chiasmata depends on the occurrence of inter-homolog crossovers (CO) during the first meiotic prophase. COs originate from the recombination mediated-repair of programmed double strand breaks (DSBs) during meiotic prophase I. Meiotic recombination differs from mitotic recombination in that it uses a chromatid from the homolog instead of the sister chromatid as a template for repair [2]. It also favors CO formation and involves specific proteins [3]. In mice, about 250–300 DSBs are generated during the leptotene stage by the catalytic activity of the conserved topoisomerase-like transesterase SPO11 [4]–[6]. DNA ends at DSBs are resected to produce single stranded DNA for homology search [7]. Only a subset of DSBs form COs, the remaining DSBs being repaired without chromosome arm exchanges. The decision to form or not a CO is thought to be made before or during strand invasion of the homologous chromosome [8]. This being the case, the nature and the regulation of proteins loaded at broken ends is likely to be important for the outcome of the DSBs. Single-strand DNA (ssDNA) formed during DNA metabolism is coated by the trimeric replication protein A (RPA) complex (composed of RPA1 70 kDa, RPA2 32 kDa, RPA3 14 kDa) serving to protect from degradation and to prevent secondary structure formation. RPA binds ssDNA with high affinity through oligonucleotide binding (OB) domains [9], [10]. During homologous recombination, RPA has to be removed from the 3′ssDNA of broken ends to allow the formation of a presynaptic nucleofilament. This is essential for homology search and the formation of a physical connection between the invading ssDNA and a homologous duplex DNA template. Whereas mitotic recombination only needs RAD51 to search and invade a homologous sequence, meiotic recombination requires an additional recombinase, the meiosis-specific DMC1 protein [11]. In plants and mammals, the presence of BRCA2, which directly interacts with RAD51 and DMC1, is necessary for their localization, however, the exact mechanisms involved in RAD51 and DMC1 loading during meiosis remain to be determined [12]–[14]. Working models based on data mainly obtained in *S. cerevisiae* and *A. thaliana* proposed that Rad51 is essential for proper Dmc1 loading and that Rad51 recombinase activity has to be inhibited to favor the homolog search bias [15]–[17].

An event tightly associated with meiotic recombination is the pairing of homologous chromosomes through the formation of the synaptonemal complex (SC). SC is a tripartite structure comprising two lateral or axial elements (AE) and a central element. Early during the leptotene stage an AE is formed along each chromosome. However, it is only after successful homology search, that complete synapsis between homologs is observed connecting the AEs of homologous chromosomes with transverse filaments at the pachytene stage [18], [19]. Subsequently, proper SC formation is required to the integrity of CO formation [20], [21].

In order to better understand the complex events that occur during meiosis, it is crucial to identify the specific proteins required for meiotic recombination. Hereby, we characterized a conserved and meiosis-specific protein containing ssDNA binding domains homologous to those of RPA1. We named this protein meiosis-specific with OB domains (MEIOB) in vertebrates. In fly, mutation of the likely *Meiob* gene homolog *hold'em* (*hdm*) reduces meiotic crossover formation and sensitizes somatic cells to DNA-damaging agents [22]. Unlike MEIOB, hdm activity is not specific to meiosis. In the present work, we demonstrate that murine *Meiob* is specifically required after DSB formation during early steps of meiosis to ensure proper DSB repair by homologous recombination, a prerequisite for efficient crossover formation and male and female fertility.

## Results

### MEIOB is evolutionarily conserved and expressed during meiosis

To identify candidate genes possibly contributing to meiosis, we performed a transcriptome analysis of magnetic-activated cell sorted (SSEA1+) male and female embryonic germ cells at 13.5 days post-coïtum (dpc). At this stage, female but no male germ cells enter meiosis. We identified *Meiob* (referred to as *RIKEN cDNA 4930528F23 gene* at the time) among the most differentially expressed genes in female germ cells (complete data set will be published elsewhere). The *Meiob* murine gene is located on chromosome 17 and is composed of 14 exons (Figure S1A) coding for a predicted protein of 470 amino acids. Amplification of the full length *Meiob* transcript from 13.5 dpc mouse ovary gave a unique band (Figure S1B), the sequencing of which confirmed the predicted sequence (data not shown).

**Figure 1:**
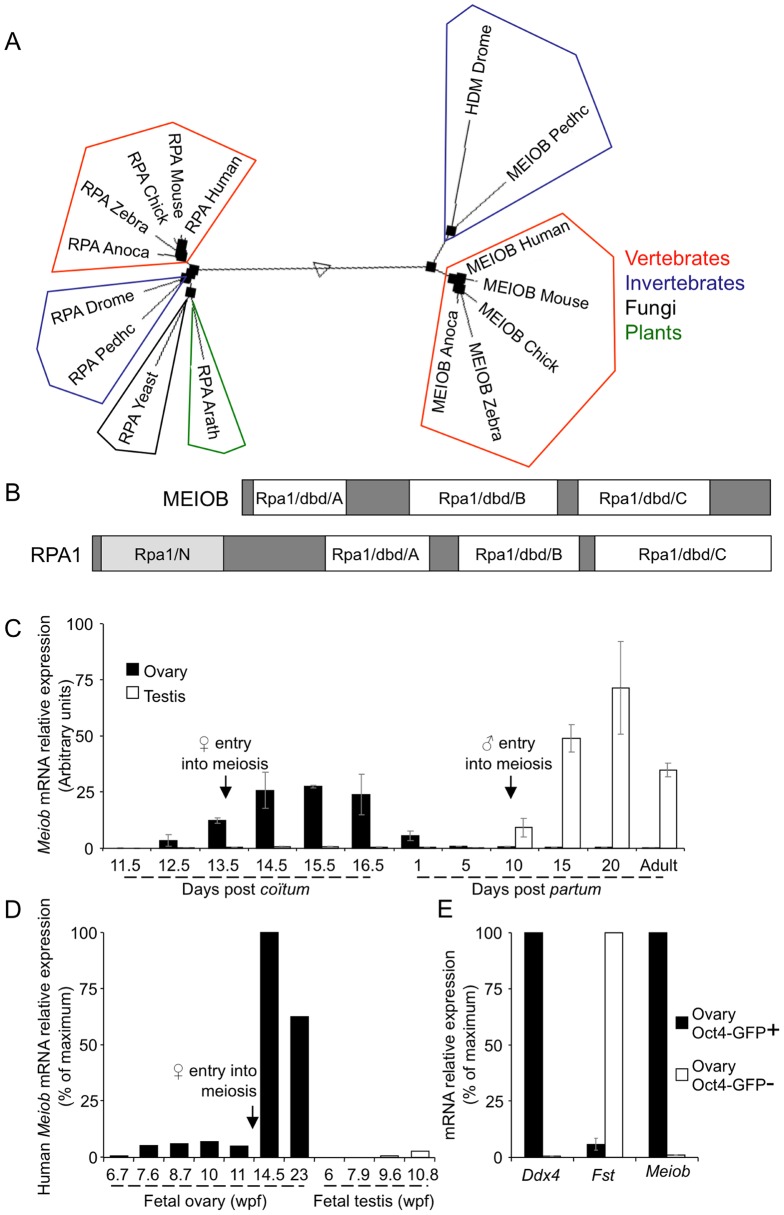
MEIOB is evolutionarily conserved and expressed in meiotic cells. (**A**) Sequences coding for putative MEIOB orthologs are found present in vertebrates and most invertebrates and are absent in fungi and plants. Multiple alignments of full length MEIOB and RPA1 protein sequences were processed with Multalin Software (http://multalin.toulouse.inra.fr/multalin/). *Meiob* and the *Rpa1* genes form distinct families. Represented species: Anoca, *Anolis carolensis*; Arath, *Arabidopsis thaliana*; Chick, *Gallus gallus*; Drome, *Drosophila melanogaster*; Human, *Homo sapiens*; Mouse, *Mus musculus*; Pedhc, *Pediculus humanus corporis*; Yeast, *Sacharomyces cerevisiae*; Zebra, *Danio rerio*. (**B**) Schematic representation of RPA1 and MEIOB protein domains. In white, three DNA binding domains (dbd) A, B and C similar to those of RPA1 are found in MEIOB protein. In light grey, RPA1 contains an N-terminal domain (RPA1N) absent in MEIOB protein. (**C**) *Meiob* expression pattern in mouse gonads. *Meiob* mRNA expression was measured using RT-qPCR in whole fetal and post-natal mouse ovaries (black column) and testes (white column). Gonads were harvested at the indicated development stages. Black arrows indicate meiosis initiation. Mean±SEM, n = 3. (**D**) *MEIOB* expression pattern in whole fetal human ovaries and testes. *MEIOB* mRNA expression was measured using RT-qPCR. Gonads were harvested at the indicated developmental stages. Wpf, weeks post fertilization. β-*actin* mRNA was used as the endogenous reporter. Data are expressed as a percentage of the maximum mRNA expression. (**E**) *Meiob* mRNA expression profile in ovary germ and somatic cells. *Ddx4*, *Fst* and *Meiob* expressions were measured using RT-qPCR in purified germ cell fraction (Oct4-GFP+, black column) and somatic cell fraction (Oct4-GFP−, white column) from mouse *Oct4-Gfp* 13.5 dpc ovaries. Mean±SEM, n = 5.

Search using tBlastn (http://blast.ncbi.nlm.nih.gov/Blast.cgi) with either the full length sequence or short conserved motifs identified *Meiob* homologs in the genomes of almost all metazoans (Figure 1A), all except *Nematoda*. Multiple alignments of full length amino acid sequences indicated a high degree of conservation of the various *Meiob* homologs in vertebrates (e.g. 91% of similarity and 85% of identity between mouse and human) while invertebrates displayed sequences with a much lower conservation (e.g. 23% of identity between mouse and fly). No ortholog could be retrieved in the genomes of fungi or plants, although a homolog was identified in the single-celled organism, *Capsaspora owczarzaki*. Interestingly, the closest *Meiob* paralog identified was the replication protein A large subunit (*Rpa1*). Consistently, InterProScan Search (http://www.ebi.ac.uk/Tools/pfa/iprscan/) identified three DNA binding domains (dbd), termed oligonucleotide/oligosaccharide binding (OB) folds, which were homologous to those of RPA1 (Figure 1B). However, the orthologs of the *Meiob* group formed a family distinct from the *Rpa1* group (Figure 1A, phylogenetic tree of MEIOB and RPA1 using MULTALIN) [23]. These data suggest that *Meiob* may have evolved from an ancestral *Rpa1* shortly before the appearance of multicellularity.

RT-qPCR analysis of *Meiob* transcription pattern in adult and fetal tissues detected *Meiob* transcripts exclusively in fetal ovary, postnatal testis and liver (Figures S2A and S2B). In the mouse ovary, *Meiob* expression started at 12.5 dpc, reached a maximum at 15.5 dpc and decreased to become undetectable in post natal life (Figure 1C). In testes, *Meiob* expression started at 10 days post partum (dpp), reached a maximum at 20 dpp and was maintained throughout adult life (Figure 1C). This expression profile was in accordance with previously published data screening genes expressed in spermatogenic cells [24]. Additionally, in human fetal gonads, *MEIOB* expression was only detected in the ovary starting at the 14^th^ weeks post fertilization (Figure 1D). Thus patterns observed in both mouse and human gonads correlate with an expression during the meiotic prophase I suggesting a conserved role within this process.

**Figure 2:**
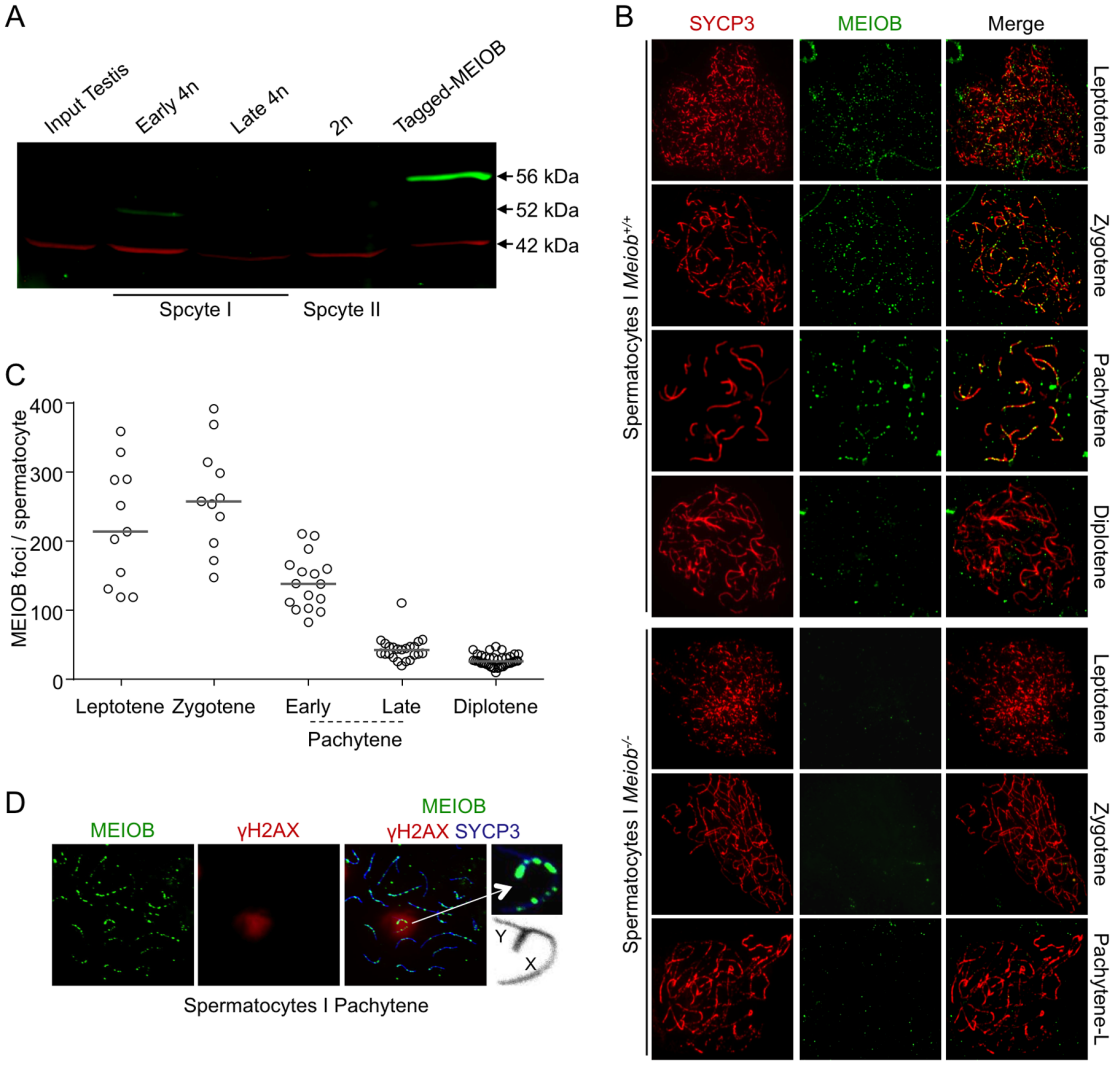
MEIOB is localized on the chromosomal axes. (**A**) MEIOB protein production in mouse adult testicular cells. MEIOB (green) and β-ACTIN (red) expression were analyzed by western blot using anti-MEIOB and anti-β-ACTIN antibodies in indicated cellular extracts. Testis cells were sorted by FACS after Hoechst 33342 staining (See Materials and Methods section and Figure S3). Early 4n: leptotene, zygotene, pachytene; Late 4n: late pachytene, diplotene; 2n: spermatocytes II; Tagged-MEIOB: HEK-293 cells transfected with *tagged-MEIOB* cDNA. (**B**) Representative chromosome spreads stained for SYCP3 (synaptonemal axial element) and MEIOB protein from *Meiob^+/+^* and *Meiob^−/−^* spermatocytes. SYCP3 staining was used to visualize the chromosome axes. MEIOB was specifically observed in wild type meiocyte spreads. (**C**) Quantification of MEIOB foci in *Meiob^+/+^* spermatocytes from adult testes. Leptotene: n = 11; zygotene: n = 11; early pachytene: n = 16; late pachytene: n = 23; diplotene: n = 40; total mice analyzed: n = 3. Median numbers of foci are marked by horizontal lines. (**D**) MEIOB, SYCP3 and γH2AX were detected in representative chromosome spreads of pachytene wild type spermatocytes. γH2AX stained sex body composed of X and Y chromosomes synapsed by pseudo-autosomal region. MEIOB foci are localized on sex chromosomes.

We next investigated which cell type expressed *Meiob* in the gonads using fluorescence activated cell sorted (FACS) germ and somatic cells from 13.5 dpc *Oct4-GFP* ovaries. *Meiob* expression levels were 112 fold higher in germ cells compared to somatic cells (Figure 1E). This germ cell-specific expression was further confirmed using *w/w* mouse fetal ovaries devoid of germ cells (Figure S2C). To determine whether the corresponding MEIOB protein was produced in these tissues, we generated two rabbit polyclonal antibodies raised against different MEIOB peptides and confirmed their specificity using a human tagged-MEIOB produced in HEK-293 cells (Figures 2A and S5A). Western blot analysis of various populations of FACS-sorted spermatocytes (Figure S3) detected a 52 kDa band consistent with the predicted MEIOB molecular weight. MEIOB protein was detected solely during early meiosis prophase I (‘early 4N’ fraction containing leptotene, zygotene and few pachytene spermatocytes) and not during later stages of meiosis (Figure 2A). No obvious signal was detected in whole adult testis protein extracts, possibly reflecting the few number of cells expressing MEIOB in this tissue (see ‘early 4N’ in Figure S3) and/or low expression levels.

### MEIOB is localized on meiotic chromosomes

MEIOB immunostaining performed on chromosomal spreads from adult testes indicated that MEIOB form foci located on chromosome axes stained with SYCP3 in spermatocytes (Figure 2B). These MEIOB foci were visible as early as leptotene stage and persisted throughout the zygotene stage, with about 250 foci per spermatocyte, and decreased drastically during the pachytene stage (Figure 2C). We observed a similar staining in 15.5 dpc oocytes, corresponding to leptotene, zygotene and early pachytene stages (Figure S4). Hardly any signal was observed on chromosome axes in spreads from *Meiob^−/−^* mice confirming the specificity of our antibodies. Signal off the axes was considered as non specific as it was retrieved in *Meiob* ^−/−^ spermatocytes and only foci on the axes were repeatedly observed when using chromosome spreads prepared with various protocols (see materials and methods section and Figure S7). At the diplotene stage, about 25 weak MEIOB foci were detected. However we cannot formally exclude that some of these foci are unspecific due to the absence of the equivalent stage in the *Meiob^−/−^* mice. During the pachytene stage, γH2AX labels the sex body containing the X and Y chromosomes that can only pair on a limited portion called the pseudo-autosomal region (PAR). Immunostaining for MEIOB and γH2AX indicated that MEIOB was retrieved on chromosomes located in the sex body at this stage and its distribution on the X appeared similar to that observed on autosomes (Figure 2D). Of note, we always retrieved at least one bright focus in the PAR and foci in the PAR were observed until mid/late pachytene stage, when few foci persisted on autosomes.

### MEIOB binds single strand DNA

**Figure 3:**
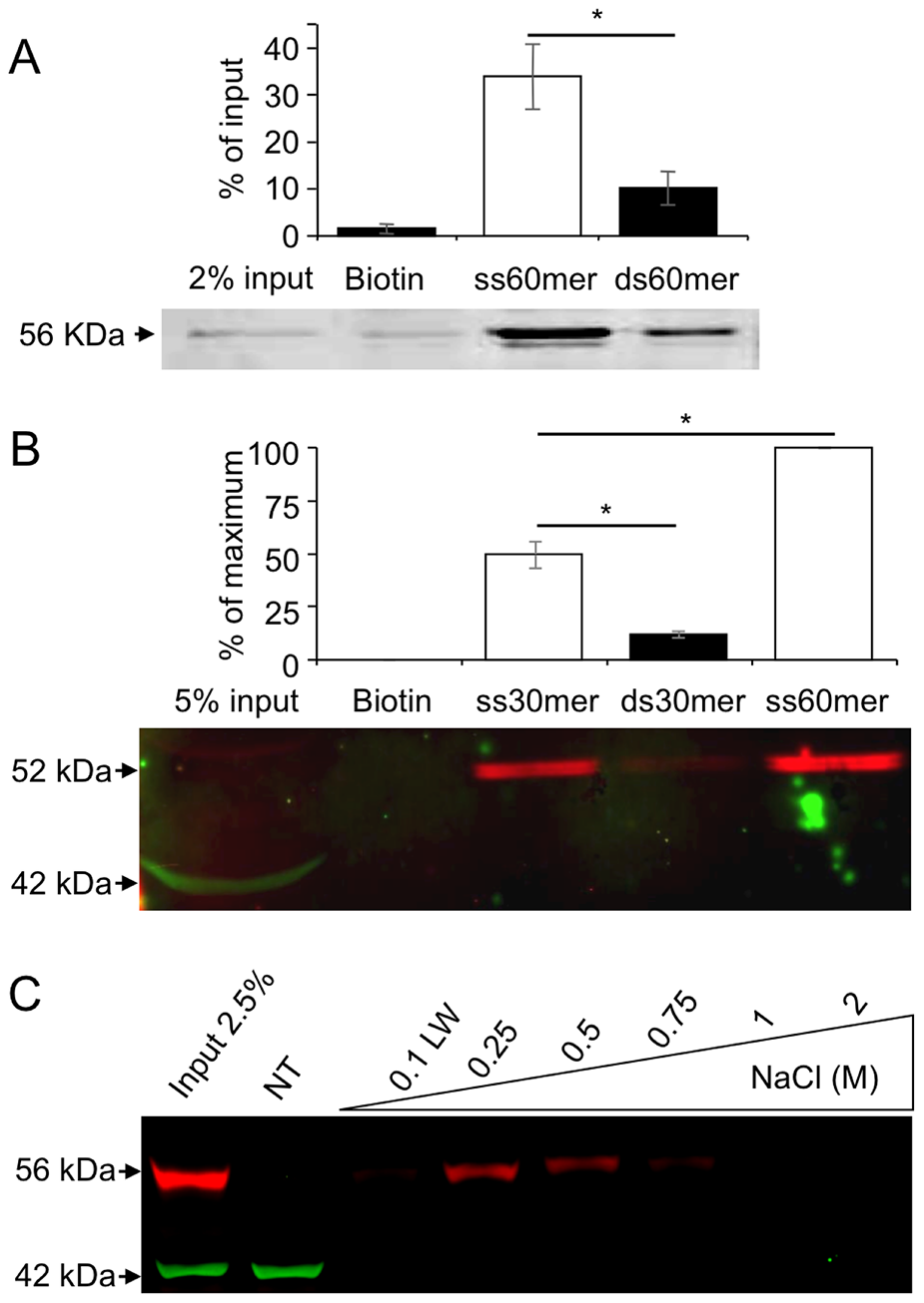
MEIOB protein binds single strand DNA. (**A**) Tagged-MEIOB expressed in a cell-free system or (**B**) testicular protein extracts were applied to beads coupled with biotin or biotinylated single strand (ss) or double strand (ds) DNA of different lengths (30 mer and 60 mer). Retained proteins were subjected to western blot hybridized with anti-β-ACTIN (green) and anti-c-MYC (A) or anti-MEIOB (B) antibodies (red). Endogenous MEIOB protein (B) and tagged-MEIOB (A) displayed respectively a size of 52 and 56 kDa, corresponding to their theoretical molecular weight. Band intensity quantifications were relative to pull down input protein extract (A) or maximal MEIOB band intensity (B) as the endogenous protein was undetectable in whole testicular protein extract. Tagged-MEIOB protein expressed in cell-free system analyzed: n = 3. Testicular protein extracts analyzed: n = 3. Mean±SEM, *, p<0.05 (paired Student's t-test). (**C**) ssDNA cellulose affinity chromatography. Whole native protein extract of HEK-293 expressing tagged-MEIOB was applied to ssDNA matrix. Bound proteins were successively eluted with the indicated NaCl solutions. First fractions of each NaCl elution were resolved by western blot hybridized with anti-c-MYC (red) and anti-β-ACTIN (green) antibodies. Controls, last eluted fractions are presented in Figure S5C. NT, non-transfected HEK-293 protein extract; LW, last wash of binding buffer.

The MEIOB being related to RPA1, a conserved ssDNA binding protein, we hypothesize that MEIOB too may bind ssDNA. To investigate whether MEIOB OB-domains confer any ssDNA binding activity, we performed ssDNA and double-strand DNA (dsDNA) oligonucleotide pull-down assays using recombinant tagged-MEIOB protein produced in a cell-free system. Pull-down assays performed with biotin 60-mer revealed a significantly greater retention of tagged-MEIOB protein on ssDNA than on dsDNA (Figure 3A). A similar experiment carried out with whole testis protein extract indicated that the endogenous protein also preferentially binds ssDNA (Figure 3B). Of note, MEIOB binding efficiency on ssDNA increased with oligonucleotide length as 30 mer ssDNA retained two fold less MEIOB than did 60 mer ssDNA. Extract from HEK-293 cells expressing tagged-MEIOB produced the same results (Figure S5B).

In order to evaluate the robustness of MEIOB binding to ssDNA, we performed ssDNA affinity chromatography. Protein extracts from HEK-293 cells expressing tagged-MEIOB were loaded on a column containing a ssDNA matrix, and proteins were eluted by progressively increasing salt concentration. Detection of eluted MEIOB by western blot revealed that MEIOB protein was eluted at a concentration of 0.75M NaCl (Figures 3C and S5C), indicating high affinity for ssDNA all be it lower than that of the trimeric RPA (most RPA being eluted at 1M and above, data not shown).

### MEIOB is localized at ssDNA sites *in vivo*

**Figure 4:**
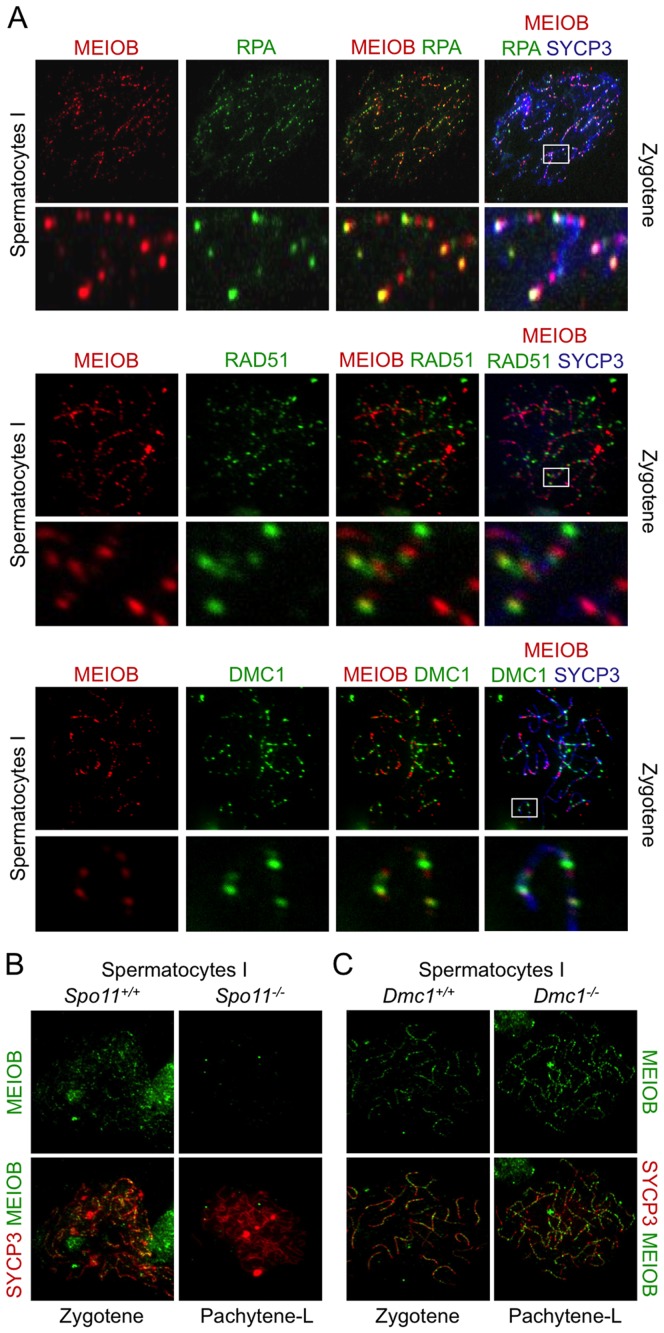
MEIOB is located at the DNA double strand breaks. (**A**) SYCP3, MEIOB and RPA2, RAD51 or DMC1 were detected in chromosome spreads of zygotene wild type spermatocytes. RPA2 and MEIOB were mostly colocalized at these stages. White squares indicate magnified regions. (**B**) MEIOB staining in *Spo11^−/−^* spermatocytes that were defective in DSB formation. Hardly any foci were observed on the chromosome axes in *Spo11^−/−^* spermatocytes. (**C**) MEIOB staining in *Dmc1^−/−^* spermatocytes that were arrested due to absence of strand invasion. MEIOB foci in *Dmc1^−/−^* appeared brighter in comparison to *Dmc1^+/+^*.

Based on our *in vitro* observations and protein domain predictions we hypothesized that MEIOB could target ssDNA *in vivo*. We therefore compared MEIOB localization to that of RPA, RAD51 and DMC1 in spermatocyte chromosome spreads. MEIOB and RPA foci overlapped considerably (Figures 4A and S6A), indicating that MEIOB is present on recombination initiation sites. However, some foci appeared solely stained for MEIOB. Respectively, 71, 76 and 86% of RPA2 foci were stained for MEIOB and 26, 49 and 61% of MEIOB foci were stained for RPA2 at leptotene, zygotene and early pachytene stages. MEIOB staining was retrieved in most RAD51 or DMC1 foci (Figure 4A), particularly in the early pachytene stage when over 80% of RAD51 or DMC1 foci were stained for MEIOB (Figures S6B and S6A). On the other hand, only about half of the MEIOB foci were stained for RAD51 or DMC1. To better characterize MEIOB recruitment we investigated its localization on chromosome spreads from *Spo11* ^−/−^ and *Dmc1* ^−/−^ mice. In the absence of SPO11, no DSBs are formed [4], [5] and we observed no MEIOB foci on the chromosome axes in spermatocytes (Figure 4B). In the absence of the DMC1 recombinase, DSBs cannot be repaired and are believed to accumulate with extensive resection [25]. As a consequence *Dmc1* ^−/−^ spermatocytes failed to complete synapsis and arrested at a stage termed ‘pachytene-like’ equivalent to late zygotene or early pachytene cells in wild type cells based on the stage of AE formation (i.e. with SYCP3 staining) [26], [27]. In *Dmc1* ^−/−^ spermatocytes, MEIOB accumulated on chromosome axes suggesting that MEIOB is recruited to the DSB sites before strand invasion (Figure 4C). Consistent with an expected localization on ssDNA and with presumed longer single stranded DNA ends at DSB sites in *Dmc1* ^−/−^ meiocytes, MEIOB staining was brighter in *Dmc1* ^−/−^ as compared to wild type spermatocytes (Figure 4C). Furthermore, in *Dmc1* ^−/−^ spermatocytes, MEIOB showed near perfect colocalization with almost all RPA2 foci (Figure S7A) and also colocalized with ATR foci (Figure S7B) a protein involved in ssDNA signaling [28].

### *Meiob* is required for fertility and meiosis completion

**Figure 5:**
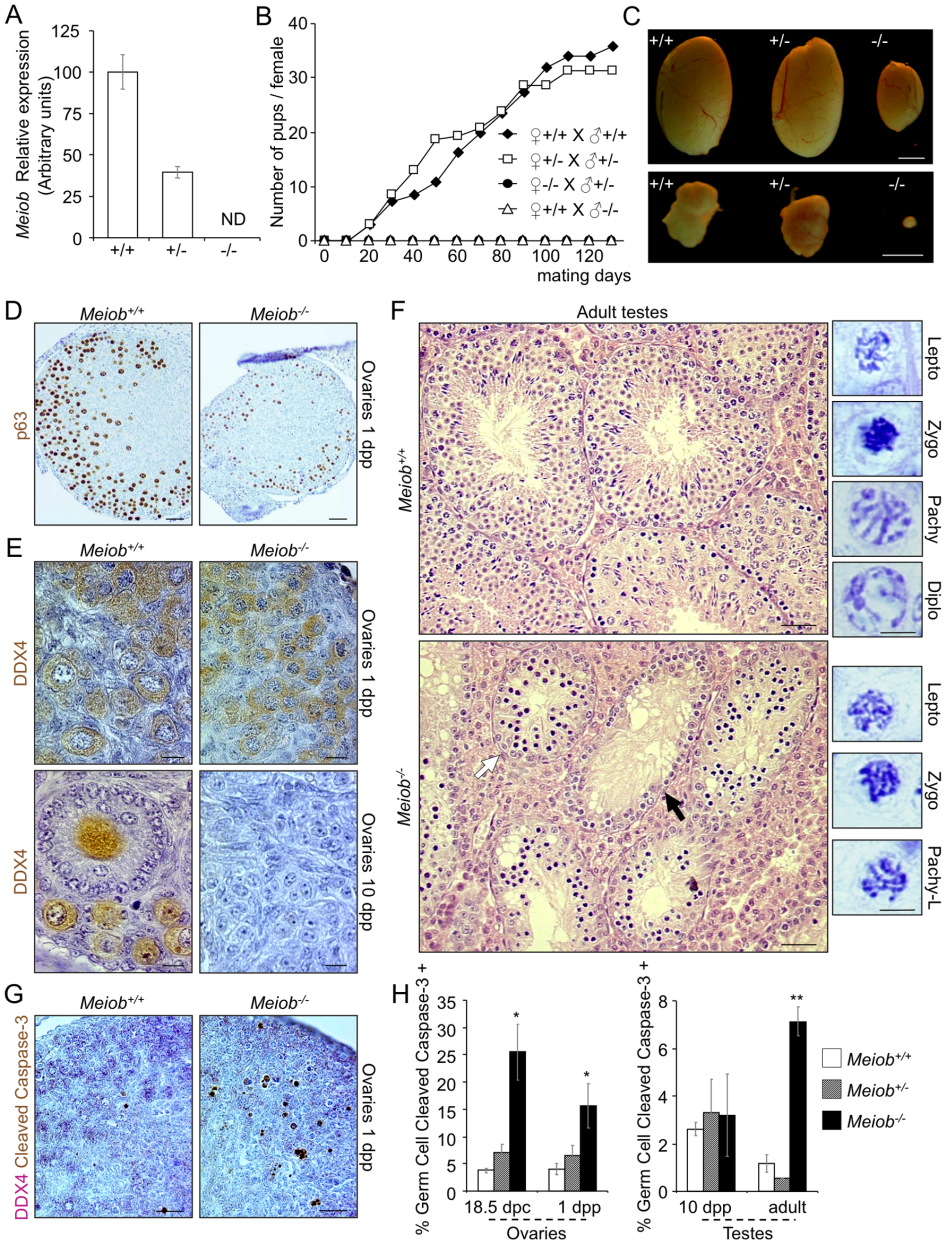
*Meiob* is required for fertility, germ cell survival and progression through meiosis prophase I. (**A**) *Meiob* mRNA is completely absent in adult testis of homozygous mutant mice. *Meiob* mRNA expression was investigated by RT-qPCR in *Meiob^+/+^*, ^+/−^ and ^−/−^ adult testes. ND, none detected. Mean±SEM; n = 2. (**B**) *Meiob* mutant homozygous mice are infertile. Mice of different genotypes were mated over a four month period and births were monitored. Values were expressed in cumulated number of pups per female (♀+/+ x ♂+/+ n = 9; ♀+/− x ♂+/− n = 7; ♀−/− x ♂+/− n = 3; ♀+/+ x ♂−/− n = 4). (**C**) Adult mice *Meiob^−/−^* testes and ovaries are strongly reduced in size by comparison with wild type gonads. Scale bars, 2 mm. (**D**) P63 immunohistochemistry in 1 dpp *Meiob^+/+^* and ^−/−^ ovaries. Scale bars, 40 µm. (**E**) DDX4 immunohistochemistry in 1 dpp and 10 dpp *Meiob^+/+^* and ^−/−^ ovaries. Scale bars, 10 µm. (**F**) Left panel. Histological sections (H&E staining) of adult *Meiob^+/+^* and ^−/−^ testes. White arrow, seminiferous tubule containing abnormal accumulation of spermatocytes; black arrow, seminiferous tubule containing only spermatogonia. Scale bars, 40 µm. Right panel. Spermatocyte chromatin morphology in adult *Meiob^+/+^* and ^−/−^ testes. Scale bars, 5 µm. (**G**) Histological sections of 1 dpp *Meiob* ^+/+^ and ^−/−^ ovaries stained for cleaved caspase-3/DDX4. Scale bar, 20 µm. (**H**) Quantification of germ cell apoptosis in *Meiob* ^+/+^, ^+/−^ and ^−/−^ testes and ovaries, respectively white, striped and black columns, at the indicated stages of development based on cleaved caspase-3/DDX4 immunohistochemistry. Mean±SEM, n = 3; *p<0.05 **p<0.001 (unpaired Student's t-test).

To investigate the role of MEIOB *in vivo, Meiob^+/−^* mice were generated through the deletion of exons 2 to 8 (Figure S1A). Transmission of *Meiob* mutant allele exhibited the expected Mendelian distribution. RT-qPCR amplifying various parts of the *Meiob* transcript (including exons 9 and 10 still present in the mutant) confirmed the complete absence of *Meiob* mRNA in the adult testis of homozygous mutants and confirmed that our genetic model was indeed a null mutant (Figures 5A and S1C). *Meiob^−/−^* mice developed and grew normally. Histological analysis revealed no anatomical defect (including in the liver, data not shown). However, *Meiob^−/−^* male and female adult mice mated for four months with *Meiob^+/−^* or *Meiob^+/+^* counterparts produced no offspring, though vaginal plugs were formed normally (Figure 5B). *Meiob* thus appears mandatory for both female and male fertility.

Next we analyzed gametogenesis in *Meiob^−/−^* mice to identify the defective steps. *Meiob^−/−^* adult testes and ovaries presented a strong reduction in size when compared to *Meiob^+/+^* gonads (Figure 5C) (testes being 3.8 times smaller (Figure S8A) and mutant ovaries hard to distinguish). During fetal life, histological analysis revealed no morphological alteration in the *Meiob^−/−^* gonads (data not shown). During post-natal life however, in 1 dpp *Meiob^−/−^* ovary, despite there being no obvious diplotene stage, the marker of late pachytene/diplotene oocytes, P63-staining, indicated the presence of numerous oocytes similar to those forming primordial follicles (Figure 5D). Germ cell counting based on immuno-detection of the germ cell marker DDX4 revealed a subsequent drop in oocyte number starting at 3 dpp (Figure S9A) until 10 dpp when no more germ cells could be observed in the *Meiob^−/−^* ovaries (Figures 5E and S9A). *Meiob^−/−^* post-natal testes histology revealed no alteration until 10 dpp. In *Meiob^−/−^* adult testes, no stage beyond primary spermatocyte was observed. Some tubules contained only spermatogonia and others contained an accumulation of spermatocytes that were mostly at the leptotene, zygotene and pachytene-like stages based on chromatin compaction and shape (Figure 5F). Furthermore, no spermatozoon was observed in *Meiob^−/−^* epididymis (Figure S8B). Cleaved caspase-3 staining indicated a significant increase of apoptotic germ cells starting after meiotic initiation in both sexes: from 18.5 dpc in *Meiob^−/−^* ovaries and from 10 dpp in *Meiob^−/−^* testes (Figures 5G and 5H). *Meiob^+/−^* male or female mice did not show any reproductive alteration when compared to *Meiob^+/+^* mice, including number of pups per litter (Figure 5B), size of adult ovaries and testes (Figures 5C and S8A), follicle population size in adult ovaries (Figure S9B), germ cell apoptosis (Figures 5G and 5H) or seminal vesicle weight (Figure S8C).

### The *Meiob* mutant displays impaired homologous synapsis

**Figure 6:**
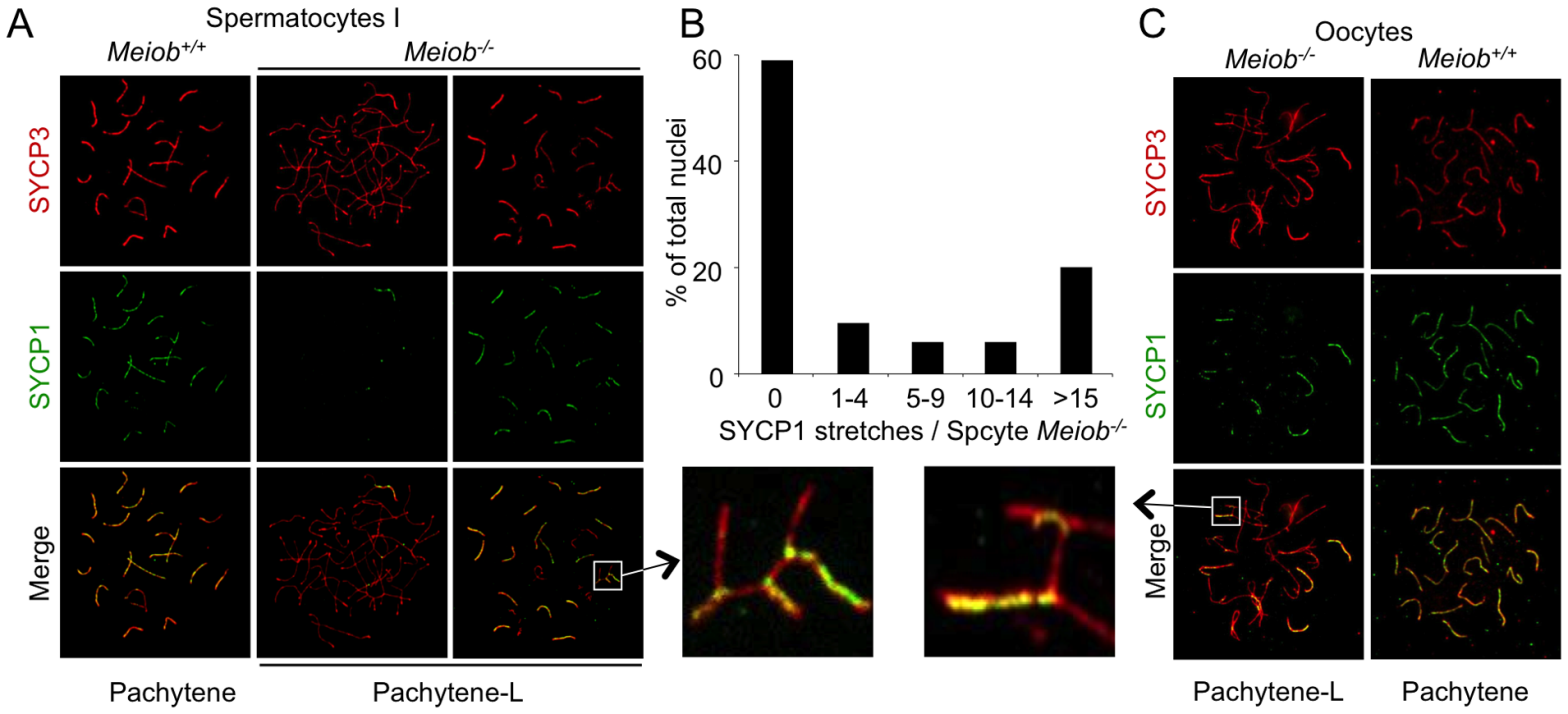
*Meiob* is necessary for the complete formation of the synaptonemal complex between homologous chromosomes. (**A**) SYCP3 (chromosome axis) and SYCP1 (synaptonemal-complex transverse filament) were detected on chromosome spreads of pachytene and pachytene-like stage spermatocytes of *Meiob* ^+/+^ and *Meiob* ^−/−^ adult mice, respectively. (**B**) Quantification of SYCP1 stretches in *Meiob* ^−/−^ spermatocytes at pachytene-like stage. Total cells analyzed: n = 85, mice analyzed: n = 3. (**C**) Representative *Meiob* ^+/+^ and *Meiob* ^−/−^ oocyte chromosome spreads at respectively pachytene and pachytene-like stages from newborn (1 dpp) ovaries, stained for SYCP3 and SYCP1. White squares indicate magnified regions that provide evidence for non-homologous synapsis in *Meiob* ^−/−^ meiocytes.

As defects in *Meiob^−/−^* occur during meiosis prophase I, we first investigated bivalent chromosome formation in wild type and *Meiob* null meiocytes. Homologous chromosomes pair and become connected along their lengths by synaptonemal complexes (SCs). AE formation is stained by SYCP3 initiated along the shared cores of sister chromatid pairs during leptotene. During zygotene a zipper like connection (stained by SYCP1) between the two AEs is initiated and completed by pachytene to fully connect homologous chromosomes. As illustrated on chromosome spreads in figure 6, the linear co-staining of SYCP3 and SYCP1 reflects fully synapsed bivalents in pachytene wild type spermatocytes. In *Meiob^−/−^* spermatocytes, as shown with SYCP3 staining, formation of AE appear to be unaffected as cells progress from leptotene to zygotene stages (Figure 2B). However, no cell with complete synapsis could be observed in adult testes (Figures 6A and 6B). The most advanced stage with regards synapsis presented an abnormal pairing of chromosomes resembling a stage between zygonema and pachynema (here termed ‘pachytene-like’ stage) (Figures 2B and 6). In such pachytene-like *Meiob^−/−^* spermatocytes, SYCP1 staining presented considerable cell-to-cell heterogeneity with regards the number of SYCP1 stretches per cell (Figure 6A). Most (∼60%) pachytene-like spermatocytes contained no SYCP1 complex and others presented from one up to nineteen (in very rare cells, less than 1%) SYCP1 stretches (Figure 6B). Similarly, 1 dpp *Meiob^−/−^* oocytes presented pachytene-like features with incomplete SYCP3/SYCP1 colocalization indicating a major defect in synapsis (Figure 6C). Both adult testes and newborn ovaries frequently displayed non-homologous pairing in *Meiob* deficient meiocytes with pairing between chromosomes of different sizes or combinations of more than two chromosomes (Figures 6A and 6C, white square magnifications).

### DNA DSB repair is impaired during meiosis in *Meiob* mutants

**Figure 7:**
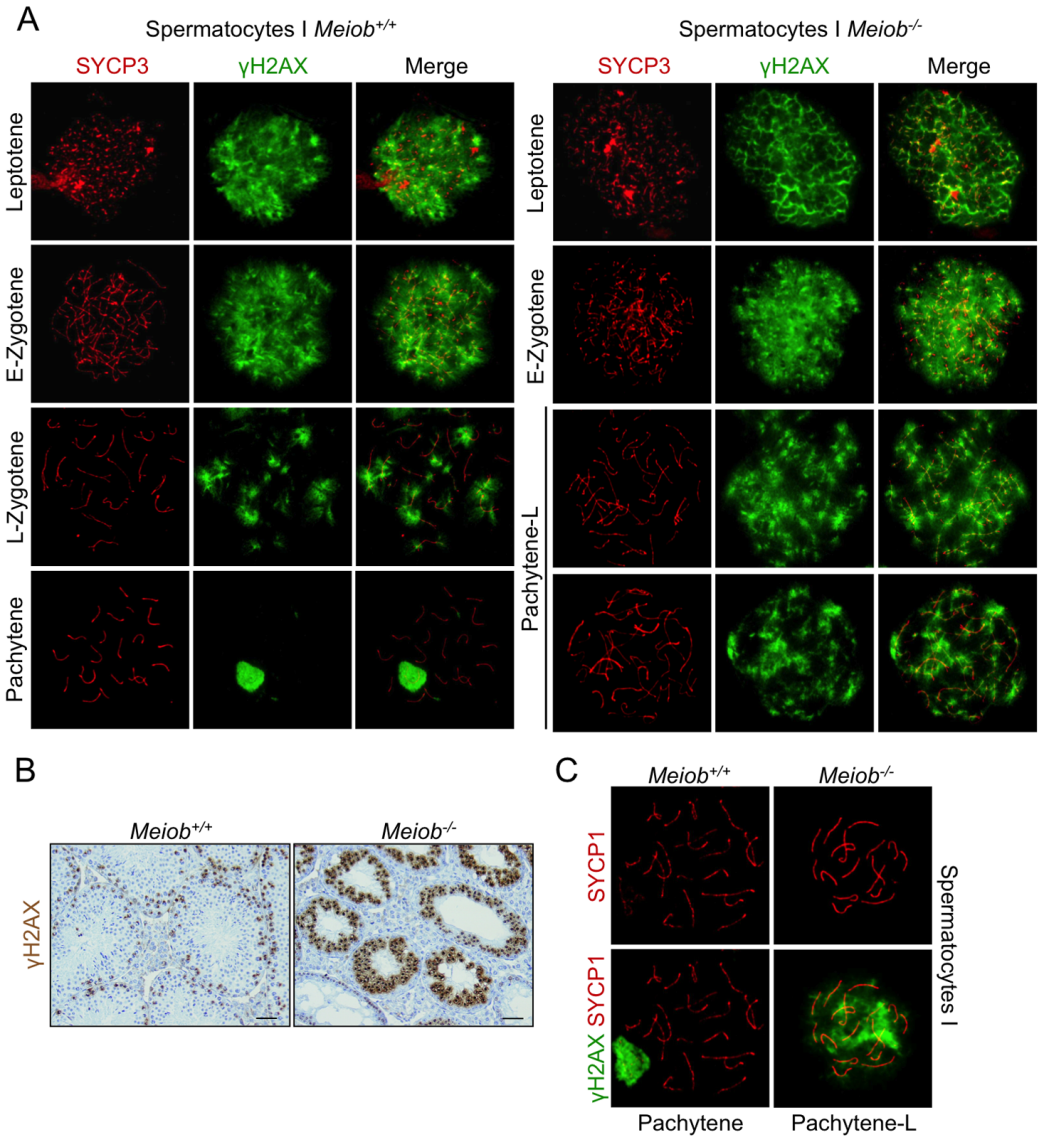
γH2AX is persistent in *Meiob* ^−/−^ spermatocytes. (**A**) *Meiob* ^+/+^ and *Meiob* ^−/−^ spermatocyte chromosome spreads at various meiosis prophase I stages, stained for γH2AX, a marker of DNA DSBs, and SYCP3. (**B**) Histological sections of *Meiob* ^+/+^ and ^−/−^ adult testis stained for γH2AX. Scale bar, 40 µm. (**C**) γH2AX and SYCP1 are detected in *Meiob* ^+/+^ and *Meiob* ^−/−^ spermatocyte chromosome spreads at pachytene and pachytene-like stages, respectively.

DSB formation and repair is essential during prophase I of meiosis. We monitored these events using γH2AX staining, γH2AX being a marker of DNA DSB [29], [30]. In sections from *Meiob^−/−^* adult testes, all spermatocytes presented a robust staining for γH2AX while such a signal is usually observed in only few cells in wild type testes (Figure 7B). Analysis of chromosome spreads of spermatocytes from wild type and *Meiob^−/−^* mice confirmed the presence of γH2AX in leptotene and zygotene cells suggesting that DNA DSBs are normally formed. In all observed chromosome spreads of pachytene-like spermatocytes from *Meiob^−/−^* mice, the γH2AX signal was maintained whereas it had disappeared as expected in pachytene spermatocytes from wild type mice (Figure 7A). Additionally, in the *Meiob^−/−^* pachytene-like spermatocytes, no structure resembling the sex body was formed (Figure 7A). In 1 dpp ovaries, wild type oocytes are mostly in late pachynema and diplonema and none are stained for γH2AX whereas *Meiob^−/−^* oocytes retained a robust γH2AX-staining at this age (Figure S10). Thus, *Meiob* appears to be required for DNA DSB repair during meiosis. In several meiotic mutants the persistence of γH2AX staining is frequently associated with synapsis defects (i.e. *Sycp*1^−/−^ [31]). Considering our finding of *Meiob* deficiency impairing synapsis, we attempted to correlate the extent of synapsis to DNA repair through SYCP1/γH2AX co-immunostaining in *Meiob^−/−^* spermatocytes. In these pachytene-like cells, even those with the highest rates of synapsis showed persistence of γH2AX staining (Figure 7C) indicating that the observed γH2AX signal is unlikely the consequence of the synapsis defect and strongly suggests persistence of unrepaired breaks.

### *Meiob* mutants are defective for meiotic homologous recombination

**Figure 8:**
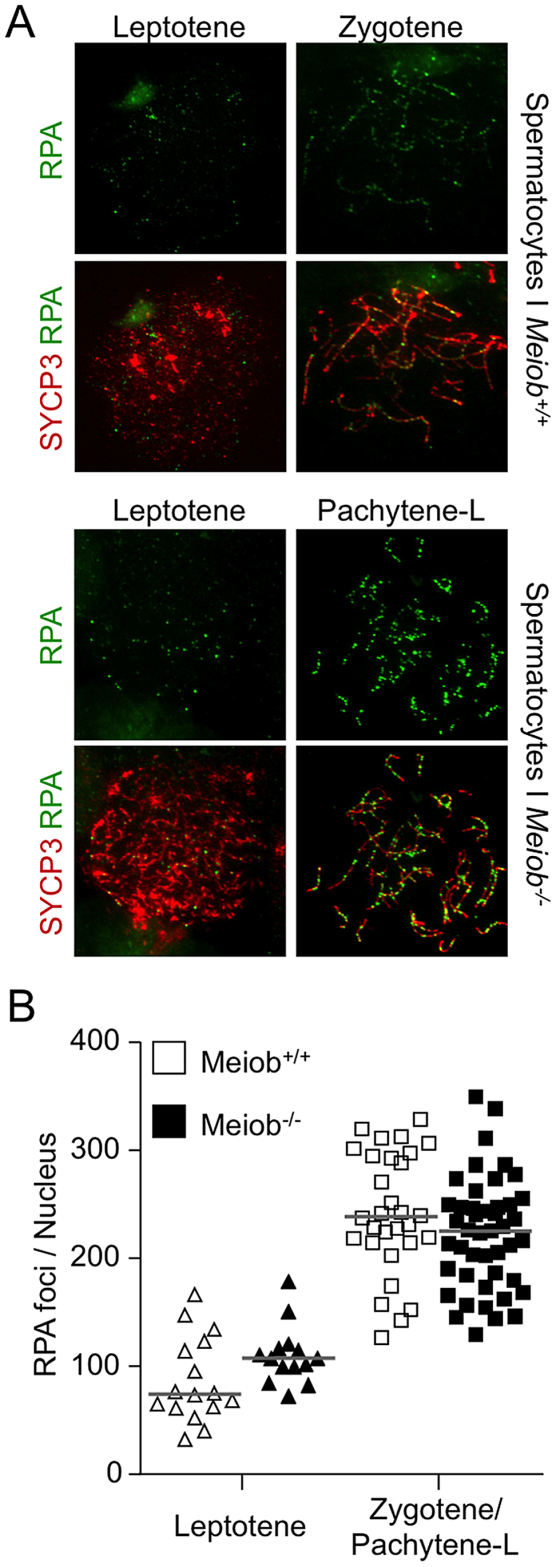
*Meiob* is not necessary for normal RPA foci number. (**A**) SYCP3 and RPA2 were detected in chromosome spreads of wild type leptotene and zygotene and *Meiob* ^−/−^ leptotene and pachytene-like spermatocytes. (**B**) Quantification of RPA2 foci in wild type spermatocytes at leptotene (n = 10) and zygotene (n = 24) stages and in mutant spermatocytes at leptotene (n = 8) and zygotene/pachytene-like stages (n = 39). Three mice were analyzed for each genotype. RPA2 foci appeared brighter in *Meiob* ^−/−^ spermatocytes by comparison with wild types. Median numbers of foci are marked by horizontal lines (unpaired Student's t-test).

**Figure 9:**
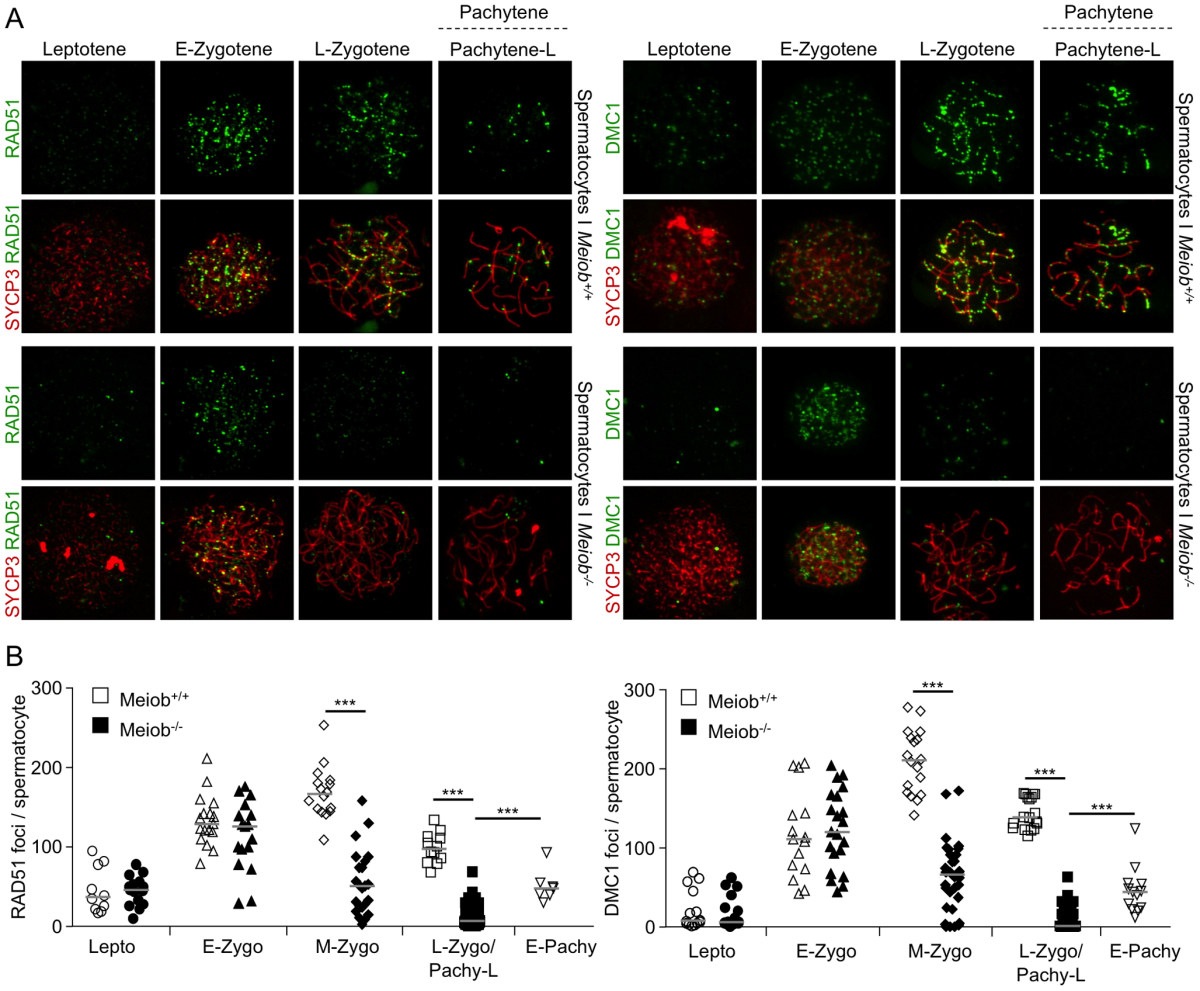
*Meiob* is necessary for the maintenance of RAD51/DMC1 recombinase foci during meiotic prophase I. (**A**) SYCP3 and RAD51 or DMC1 were detected in chromosome spreads of leptotene, early zygotene, late zygotene, and pachytene/pachytene-like spermatocytes from *Meiob* ^+/+^ or ^−/−^ mice using antibodies that recognized specifically RAD51 or DMC1. RAD51 and DMC1 foci appeared at leptotene stage and disappeared prematurely during the zygotene stage in *Meiob* ^−/−^ spermatocytes. (**B**) Quantification of RAD51 and DMC1 foci in *Meiob^+/+^* spermatocytes at leptotene (Lepto; n = 10 and 14), early-zygotene (E-Zygo; n = 17 and 15), mid-zygotene (M-Zygo; n = 16 and 18), late-zygotene (L-Zygo; n = 12 and 18) and early-pachytene (E-Pachy; n = 7 and 13) stages and in and *Meiob^−/−^* spermatocytes at leptotene (n = 15 and 16), early-zygotene (n = 17 and 21), mid-zygotene (n = 23 and 32) and pachytene-like (Pachy-Like; n = 48 and 43) stages. Median numbers of foci are marked by horizontal lines; ***p<0.0001 (unpaired Student's t-test).

**Figure 10:**
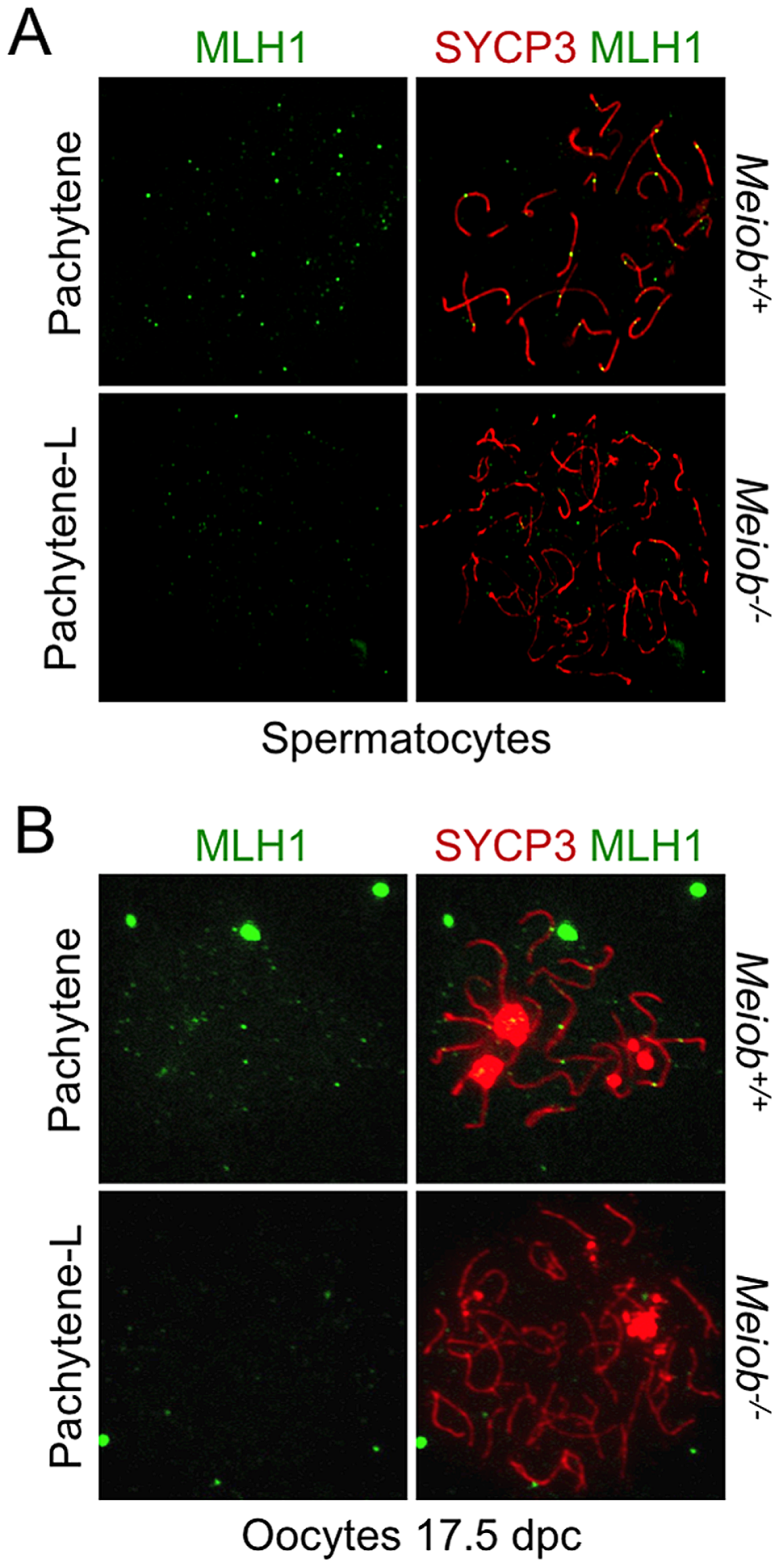
*Meiob* is necessary for CO formation. MLH1 and SYCP3 were stained in *Meiob* ^+/+^ pachytene and *Meiob* ^−/−^ pachytene-like spermatocyte (**A**) and oocyte (**B**) nuclear spreads. MLH1 foci were undetectable along axial elements in *Meiob* ^−/−^ meiocytes.

Taking into account MEIOB spatial and temporal localization and its requirement for proper DSB repair, we investigated homologous recombination in *Meiob^−/−^* spermatocytes through immunolocalization of RPA2, RAD51, DMC1 and MLH1. In *Meiob^−/−^* leptotene and zygotene/pachytene-like cells, RPA foci were formed on chromosome axes and in normal abundance when compared to wild type equivalent stages (Figures 8A and 8B). This equally confirms that DNA DSBs were produced with no overt defect in the absence of MEIOB. Immunolocalizations of RAD51 and DMC1 were performed with antibodies recognizing specifically RAD51 or DMC1. In *Meiob^−/−^* leptotene stage spermatocytes, RAD51 and DMC1 foci were localized on chromosome axes with no overt differences in comparison to wild type counterparts (Figure 9). In wild type zygotene stage these foci were maintained and their numbers decreased over the course of the pachytene stage. However in *Meiob^−/−^*, we observed a massive decrease in both RAD51 and DMC1 stainings at zygotene and pachytene-like stages. By comparison with wild type, the number of RAD51 and DMC1 foci respectively decreased by 70% and 69% at mid-zygotene in *Meiob^−/−^* (Figure 9B). Measurement of RAD51 and DMC1 foci intensities indicated no significant change in the mean intensity during leptotene and zygotene stages (Figure S11A). Intense (high and medium) foci tended to decrease first in mid-zygotene and all foci intensely and mildly (low) stained considerably decreased in late zygotene/pachytene like stage in Meiob^−/−^ spermatocytes (Figure S11B). These data indicate that while the formation of RAD51 and DMC1 foci is unaltered in *Meiob^−/−^* mutants, their stabilization is impaired. The absence of synapsis, the persistence of γH2AX and the reduction of RAD51 and DMC1 foci observed in the absence of MEIOB suggest a strong defect in recombination with consequential impairment of CO formation. To test this hypothesis we investigated CO formation using MLH1 immunostaining. MLH1 is believed to mark future CO sites in mid-to-late pachytene, and is essential at the late stages of recombination in the formation of CO [32]. As expected at least one MLH1 focus was observed per bivalent chromosome in wild type pachytene spermatocytes. In contrast, no MLH1 foc1 were observed in *Meiob^−/−^* spermatocytes (Figure 10A). This likely reflects a blockade prior to mid-pachytene preventing thus CO formation. *Meiob* ^−/−^ oocytes are eliminated at a developmental time-point where most wild type oocytes reached a stage beyond pachytene. This allowed us to address whether MEIOB is required or not for the formation of MLH1 foci in cells that likely correspond to pachytene stage based on the developmental time-course of wild type cells. Many oocytes are in pachytene stage, and have MLH1 foci in wild type 17.5 dpc embryos. Interestingly we were unable to observe bright MLH1 foci along the AEs in the oocytes of 17.5 dpc *Meiob^−/−^* embryos (Figure 10B). Altogether these data suggest that MEIOB may be involved in both early/homology search –related and possibly also in later/post-homology search steps of meiotic recombination and CO formation.

## Discussion

In this study, we have identified a new major player in mammalian meiosis and in particular have demonstrated for the first time an absolute requirement of MEIOB for meiosis prophase I completion in the mouse. The expression profile and deletion of *Meiob* indicated a specific role for this protein during meiosis prophase I. Furthermore, our data indicate that MEIOB is mandatory for DSB repair and crossover formation. MEIOB also appears to favor faithful and complete synapsis. As observed with other mutant mice in which these steps are impaired, these defects caused meiotic arrest, meiocyte death by apoptosis and infertility. The complete infertility of both male and female mice indicates that *Meiob* is one of the core genes required for meiosis, as the deletion of other meiotic genes in mice sometimes only induces male infertility [12], [34]–[35]. In summary, this mammalian meiotic mutant impairs meiotic recombination in both male and female and impairs RAD51 and DMC1 stabilization.

Our data support the idea that MEIOB binds ssDNA. First, protein domain prediction proposed that MEIOB contains three ssDNA binding domains, denoted OB domains, similar to those of RPA1 known to have a high affinity for ssDNA [36]. Second, *in vitro* DNA binding assays demonstrated that MEIOB is particularly retained by ssDNA. Moreover, MEIOB foci formed along AE and colocalized to a large extent with RPA which marks ssDNA filament [37], [38]. The number of foci corresponded to the expected number of DNA DSBs generated by SPO11 and no MEIOB foci were observed in the absence of SPO11-mediated DSBs. Lastly, when 3′ssDNA accumulated (i.e. in *Dmc1* ^−/−^, in the absence of strand invasion) MEIOB colocalized almost perfectly with both RPA and ATR, known to sense ssDNA [28], [39]. Altogether these results lead us to propose that the MEIOB protein binds ssDNAs generated during the 5′ to 3′ resection of DNA ends at meiotic DSB sites. We thus propose that MEIOB may be an RPA paralog specifically required for meiotic recombination. Although the data in the present study do not formally prove that the ssDNA binding activity of MEIOB is required for meiosis, this is an appealing hypothesis that needs pursuing in future studies.

*Meiob^−/−^* meiocytes are arrested during prophase I of meiosis at a stage resembling late zygotene stage and characterized by persistent DSBs and defects in chromosomal pairing. This incomplete synapsis is likely the consequence of the defective DSB repair as is observed in most mutants with impaired homologous recombination [12], [26]. In *Meiob^−/−^* meiocytes the number of DSBs appears unaffected as we observed the expected number of RPA foci [38] and the RAD51 and DMC1 foci were formed but did not persist. These observations point towards the lack of MEIOB primarily causing a defect in the process of recombination. Unfortunately, due to their embryonic lethality, most mutations that impair homologous recombination have not been characterized during mammalian meiosis. Notably, the lethality of *Rad51* ^−/−^ disallows the comparison of *Meiob* ^−/−^ to these murine meiocytes. Of note, we would like to point out that *Meiob* ^−/−^ phenotype is similar to that of *Dmc1* ^−/−^ mice [26], [27]. However in the *Dmc1^−/−^* spermatocytes, RAD51 persists in the pachytene-like stage in contrast to that observed in *Meiob^−/−^* mice ([26], Figure S12). Interestingly, in *Meiob^−/−^* pachytene-like cells, despite the absence of RAD51 and DMC1 foci, we observed abundant and brighter RPA foci and strong γH2AX staining indicating the presence of unrepaired DSBs and of ssDNA. We thus conclude that the transitory loading of RAD51 and DMC1 observed in *Meiob^−/−^* is insufficient to allow the completion of homology search, homolog alignment and SC formation. As RAD51 recombinase activity is believed to be inactivated during meiotic recombination, DMC1 is proposed to catalyze homology search and strand exchange of most meiotic recombination events [40]. Thus, in the *Meiob* mutant, DMC1 would appear to have been prevented from performing its role due to its precocious destabilization. To our knowledge, no murine meiotic mutant has yet presented such a phenotype with a premature disappearance of RAD51 and DMC1 before pachynema.

The formation of RAD51-DNA presynaptic filament is promoted by BRCA2 in mammals to overcome the inhibitory effect on the heterotrimeric RPA [41]. BRCA2 is known to bind both RAD51 and DMC1; *Brca2* ^−/−;Tg^ have an impaired number of RAD51 and DMC1 foci in leptotene and zygotene mouse spermatocytes [12]. Thus the *Meiob^−/−^* defect does not seem to involve BRCA2 in a general manner as one would then have also expected an earlier defect in the loading of RAD51 and DMC1. Moreover, the growing percentage of RAD51 and DMC1 foci that are stained for MEIOB at late zygotene and early pachytene stages support a later role of MEIOB on the activity of recombinases. In this line, biochemical findings raise the possibility that the maintenance of RAD51 presynaptic filament *in vivo* might involve some RAD51 accessory factors such as RAD54, HOP2-MND1 and recently the SWI5-SFR1 complex [42]–[44]. The spermatocyte defects observed in *Rad54^−/−^* and *Hop2^−/−^* mice differ from those observed in the *Meiob* mutant. Indeed meiotic recombination is only slightly affected in *Rad54^−/−^* mice which in turn show no major fertility defect [45]. In *Hop2^−/−^* mice, despite a failure of meiotic recombination, RAD51 and DMC1 foci accumulate and persist through the pachytene-like stage [46]. The role of SWI5-SFR1 has to date only been investigated in mouse embryonic stem cells [47], however genetic studies in yeast have found evidence for the SWI5-SFR1 yeast orthologs Mei5 and Sae3, being involved in meiotic recombination [48], thereby suggesting that the mammalian SWI5-SFR1 may also play a role in meiotic recombination. Interestingly, this complex is devoid of DNA-binding activity in mice [49] whereas their respective orthologs Mei5 and Sae3 in *S. cerevisiae* possess DNA-binding activity [50]. Furthermore, the expression of Mei5 and Sae3 is restricted to meiosis and mediates Dmc1 activity by enhancing its ability to form nucleofilaments on ssDNA [51]. One may reasonably speculate therefore that MEIOB may influence the stability of the RAD51-DMC1 filament in cooperation with SWI5-SFR1 or a meiotic counterpart. Such a hypothesis will of course require further investigation. Of note, SWI5-SFR1 and Mei5-Sae3 complexes directly interact with RAD51 [49], [50]. We were unable to detect a direct interaction between MEIOB and RAD51 (data not shown). However our experiments were performed in a heterologous system and with an overexpressed protein, thus we cannot formally allow us to exclude a direct interaction between MEIOB and RAD51. Finally, we propose that the presence of two ssDNA binding proteins, namely RPA and MEIOB, confers special properties to resected DNA allowing the proper stabilization of two proteins on the meiotic presynaptic filament: DMC1 and RAD51. Along this line, one may consider MEIOB as a new meiotic-specific mediator for RAD51/DMC1.

While meiosis recombination is a conserved process in eukaryotes, there are clear differences among organisms [52]. Interestingly, yeast, plants and *C. elegans* do not have a *Meiob* homolog (Figure 1A). One may therefore consider that in these organisms, either meiosis is achieved via slightly different mechanisms or the function of MEIOB is performed by other proteins. For instance, in plants, there are multiple copies of RPA1 (RPA1-like proteins) [53], [54] some of which may assume the role of MEIOB. In plants, RPA1-like proteins have been proposed to form part of trimeric RPA complexes alongside RPA2 and 3 sub-units [55]; to our knowledge no such additional sub-units are conserved among vertebrates and the analysis of transcriptomic data did not allow us to pin point any meiosis-specific additional RPA2-like protein in mice. We therefore propose that either MEIOB acts with the canonical RPA2 and 3 (thus replacing RPA1) or by its own and possibly with different partners. The only *Meiob* homolog that has been described previously is *hold'em* (*hdm*) in Drosophila [22] that is implicated in crossover formation. However *hdm^−/−^* fly and *Meiob* ^−/−^ mice present drastic divergence during meiosis suggesting that their function may have diverged during evolution. Indeed in *hdm* ^−/−^ flies, some crossovers still occur though with lower frequencies compared to wild-type fly and the DSBs are repaired though with a delay. Furthermore, hdm is also involved in DNA repair in somatic cells [22] while *Meiob* is almost exclusively expressed in meiotic germ cells. Joyce *et al.* also provided evidence that hdm physically interacts with Ercc1, a member of the exchange class of gene products, and proposed that the Ercc1/Mei9/Mus81/hdm complex functions in the meiotic recombination pathway to resolve DSB-repair intermediates into crossovers [22]. This function appears to be required later by comparison to the here-described recruitment of MEIOB to DSB sites. Furthermore, such an interaction is unlikely to explain the entirety of *Meiob^−/−^* phenotype described here. Indeed, in mice, *Ercc1* deletion does not prevent synapsis or sex body formation and males are able to produce few spermatozoa albeit with DNA damage [56]. We thus conclude that, in mice, the function of MEIOB is not through an interaction with ERCC1 in late recombination nodules and that additional partners need to be identified. On the other hand, one could also argue that this may be due to the meiotic process itself having slightly different requirements in-between mammals and drosophila. Therefore, we cannot exclude an additional role of MEIOB in CO resolution. Such a role could be responsible of the late staining observed for MEIOB (Figures 2B and 2C). In this line we could speculate that MEIOB might target additional ssDNA sites such as the D-loop intermediate, as it is thought for RPA [57], or the second end of the DSB (i.e. the one not involved in the initial homology search), that are formed during the process of CO. The second end is released to be captured on the recombination intermediate to form a double holiday junction (dHJ). This would fit with our reports of rare cells displaying some amount of pairing, thus having performed the initial homology search (first end), and with the complete lack of MLH1 foci observed in *Meiob^−/−^* cells. Such a speculative proposition might also be fueled by the persistent colocalizsation of MEIOB with recombinases and will require additional studies. Putative MEIOB partners are under investigation and their identification should help define the precise function of MEIOB during meiotic recombination and the mechanisms allowing recombinase stabilization in this process.

## Materials and Methods

### Mice and embryos

All animal studies were conducted in accordance with the guidelines for the care and use of laboratory animals of the French Ministry of Agriculture. Mice were raised and mated, and fetal gonads were isolated as previously described [58], [59]. Mice used in this study were NMRI mice (Naval Maritime Research Institute), *Dmc1* and *Spo11* mutant mice, *w/w* mice, *Oct4*-GFP mice (that have been previously described, respectively [5], [26], [60], [61]) and *Meiob* mutant mice (see below).

### *Meiob* mutant allele construction

The *Meiob* null allele was established at the Mouse Clinical Institute/Institut Clinique de la Souris (MCI/ICS), Illkirch, France (http://www.ics-mci.fr/). The targeting vector was constructed as follows: a 4.5 kb fragment encompassing exon 2 and part of the first intron, *Red Fluorescent Protein* (*Rfp*) cDNA, followed by a neomycin resistance cassette surrounded by two loxP sites and a 4 kb fragment encompassing exons 9 and 10 (Figure S1A). *Meiob* endogenous ATG in exon 2 was conserved to ensure *Rfp* cDNA expression and there was a stop codon at the end of *Rfp* cDNA (Figure S1A). The linearized construct was electroporated in BD10 C57BL/6J mouse ES cells. After selection, targeted clones were identified by PCR using external primers and further confirmed by Southern blot with a Neo probe, 5′ external probe and 3′ external probe (data not shown). Two positive ES clones were injected into 129/Sv blastocysts and the derived male chimaeras produced germ-line transmission. *Meiob^+/Rfp-loxPNeoloxP^* mice were crossed with mice carrying ubiquitous *Cre* in order to remove the Neo resistance cassette and generate the final *Meiob* null allele (Figure S1A). Mice were genotyped by PCR of tailed DNA using REDExtract-N-Amp Tissue PCR Kit (Sigma) according to the manufacturer's instructions. Sequences of primers used are shown in Table S2.

### Fertility assay

Male and female *Meiob* ^+/+, +/− & −/−^ mice were mated over the course of four months. Mating partners were inverted every two months. Vaginal plugs were regularly checked to verify that *coïtum* occurred normally. Births and pups were counted and referenced every day and pups were sacrificed. Results are expressed in cumulative numbers of pups per couple per ten days.

### Collection of human fetal gonads

Human fetal material was provided by the Department of Obstetrics and Gynecology at the Antoine Béclère Hospital, (Clamart, France) following legally induced abortions in the first trimester of pregnancy and therapeutical termination of pregnancy in the second trimester. Human fetal gonads were harvested as previously described [62]. Our study was approved by the Biomedicine Agency (reference number PFS12-002), and all women gave their informed consent.

### Generation of anti-MEIOB antibody

Rabbit polyclonal anti-MEIOB antibodies were generated and affinity purified by Eurogentec (Angers, France) using double X protocol with ADP TEA SRN LAR QGH T and IRE NKE TNV ADE IDS polypeptides.

### Flow cytometric characterization of MEIOB localization

Testicular single-cell suspensions and cell sorting were processed as previously described [63]. Dissociation was performed with two wild type adult testes resulting in the sorting of two hundred million testicular cells. Seven hundred thousand cells from each sorting gate were lysed and submitted to western blot analysis.

### Real-time quantitative PCR and PCR

RNA extractions and RT-qPCR were performed as previously described [64]. Sequences of primers are shown in Table S2. β*-actin* mRNA was used as the endogenous reporter. Data are expressed as a percentage of the maximum mRNA expression unless otherwise stated in which case an external reference (F9 cells) was used to normalize the expression of numerous points.

### Cell culture and plasmid transfection

Human Epithelial Kidney cells (HEK-293, ATCC) were cultivated in DMEM High Glucose (Gibco) containing 15% FSB (Gibco). Human *MEIOB* complete cDNA was inserted into a pCMV6-Entry plasmid (Origene cat# RC228391) containing c-Myc and Flag tags in the C-terminal domain of the protein. This plasmid was transfected into HEK-293 cells using Lipofectamine 2000 (Invitrogen) according to the manufacturer's instructions. Tagged-MEIOB was observed in both nuclear and cytoplasmic compartments of the transfected HEK-293 cells (Figure S5A).

### Protein extraction and recombinant protein production

Protein extracts were produced from cell lines or tissues under native conditions. HEK-293 cells were harvested, centrifuged 5 min at 500 g and then lysed in Cell Lysis Buffer (Cell signaling, Danvers, USA) complemented with 1 mM 2-mercaptoethanol and Complete protease inhibitor (Roche, Mannheim, Germany). For adult testis, the albuginea was first removed and testis was lysed in the same buffer using ceramic spheres and FastPrep-24 Instrument (MP Biomedicals) with two pulses of 20 seconds. Extracts were then gently sonicated (two pulses of 30 seconds), centrifuged 10 min at 14 000 g and supernatant was immediately used for functional applications. Human recombinant tagged-MEIOB protein was produced in a cell-free system. The TnT T7 Quick Coupled Transcription/Translation System (Promega) was used with 1 µg of the tagged-MEIOB plasmid in a 50 µl reaction according to the manufacturer's instructions.

### Western blotting

Prior to gel migration, protein samples were supplemented with Laemmli buffer and resolved by 10% SDS-polyacrylamide gel electrophoresis (SDS-PAGE). Gels were electrophoretically transferred to polyvinylidene difluoride membranes (PVDF) (Amersham Biosciences, Buckinghamshire, England) before hybridization with appropriate primary antibodies and fluorescent dye coupled secondary antibodies (See Table S1). Images were acquired using Typhoon 9400 scanner (Amersham Biosciences) and quantified with ImageJ software [65].

### Histology, immunohistochemistry and germ cell counting

Histological sections, germ cell counting and immunohistochemical stainings using appropriate antibodies (Table S1) were performed as previously described [59].

### Preparation of meiocyte chromosome spreads

Testes used for spermatocyte spread preparation were harvested from mice aged approximately 2 to 6 months. Ovaries used for the preparation of chromosome spreads were harvested in 15.5 and 17.5 dpc embryos and 1 dpp pups. Three types of meiocyte chromosome preparations were used. For γH2AX, SYCP1 and RPA2 stainings, chromosome spreads were prepared according to the drying-down technique [66] with minor, previously described modifications [67]. For RAD51, DMC1 and ATR stainings, chromosome spreads were prepared as previously described [68]. This later protocol was also used for MLH1 staining in oocytes and the former protocol was used for MLH1 staining in spermatocytes. For MEIOB staining, the two above protocols were used and an additional spermatocyte surface-spread technique was applied as follows. Spermatocytes were dispersed in an 80 mM NaCl drop, attached to glass slides, fixed with a 4% paraformaldehyde 0.03% sodium dodecyl sulfate solution and washed in 0.4% Photo-Flo 200 (Sigma). RAD51 and DMC1 antibodies were previously published [69]. The specificity of the DMC1 antibody was further validated using Dmc1^−/−^ spermatocytes and no signal was retrieved (data not shown). Due to the lack of Rad51^−/−^ spermatocytes (i.e. Rad51 deletion is lethal), no such control was performed for the RAD51 antibodies and one cannot formally exclude that these might crossreact.

### Immunofluorescence on preparations of meiocyte chromosome spreads

Spermatocyte and oocyte preparations were washed with 0.4% Photoflo 200 (Sigma) and slides were air-dried 15 minutes before permeabilizing and blocking. They were then incubated overnight with the appropriate primary antibodies in blocking solution at room temperature, followed by 1 hour incubation with secondary antibodies at 37°C (see Table S1 for antibodies). Slides were mounted with Vectashield DAPI medium (Vector Laboratories). Imaging was performed using a AX70 epifluorescent microscope (Olympus) equipped with a charge-coupled camera (Roper Scientific) and IPlab software (Scanalytics) or with a DM5500 B epifluorescent microscope (Leica Microsystems) equipped with a CoolSNAP HQ^2^ camera (Photometrics) and Leica software (Metamorph). Images were processed and specific structures were quantified with ImageJ software (Cell Counter and Foci Picker3D programs). For quantification of foci colocalization, foci overlapping for the most part were considered as colocalized without restriction to foci that share the same centroid (e.g. immediately adjacent foci were not considered as colocalized).

### ssDNA/dsDNA pull down assay

HPLH purified biotinylated oligonucleotides were used for the DNA pull down assays: ss30-mer 2: 5′-GAT CTC AGC GAT TCA CAC GCG TCC TAA CTC G-3′-BiotinTEG, ss60-mer: 5′-GAT CTG CAC GAC GCA CAC CGG ACG TAT CTG CTA TCG CTC ATG TCA ACC GCT CAA GCT GC-3′-Biotin TEG (Eurogentec). Double strand hybridizations were performed in 50 mM NaCl, 25 mM Tris-HCl, pH 7.5 buffer with complementary sequences at molecular equivalence by a denaturing step (3 minutes 94°C) and a slow progressive return to room temperature. 0.2 pMol of DNA was immobilized onto 1 µg Dynabeads M-280 Streptavidin (Dynal) following the protocol supplied by the manufacturer. Protein extracts were pre-incubated on ice for 10 minutes in modified DBB (DBB with 25 mM Tris-HCl, 1 mM EDTA plus 5 mg/ml BSA for the cell-free protein assays or plus 10 µg/ml BSA for assays with total protein extracts) before addition of 500 µg Dynabeads with immobilized ss- or ds-DNA probes. DNA binding assays were conducted either with 3 µl of the tagged-MEIOB cell-free productions, with 600 µg of protein from HEK-293 cells transfected with h*MEIOB* (Figure S5A) or with 3 mg testis protein extracts. The DNA-protein mixture was incubated for 1 hour at 4°C under gentle agitation. After magnetic separation, the beads were washed three times in 500 µl binding buffer without BSA, before being washed once in 500 µl rinsing buffer (modified DBB with 150 mM NaCl). Elution of DNA binding proteins was performed by resuspending the beads in 20 µl Laemmli buffer and boiling the samples for 5 minutes before magnetic separation of the beads and western blotting of the samples.

### ssDNA-cellulose affinity chromatography experiments

HEK-293 cells were harvested 48 h after transfection of the *tagged-MEIOB* plasmid. Three mg of total protein extract were diluted 1/5 in DNA-Binding Buffer (DBB: 50 mM Tris-HCl, 100 mM NaCl, 10% (w/v) glycerol, Complete Protease Inhibitor, 1 mM 2-mercaptoethanol, pH 7.4), 1 ml of which was loaded in Poly-Prep columns (Biorad), previously loaded with 500 µl of ss-DNA-cellulose (Amersham Biosciences). Protein extract was incubated 90 min at 4°C under gentle agitation. Columns were washed with forty bed volumes of ss-DBB and the last 1 ml of wash was kept to ensure that complete elution occurred (0.1M LW). Columns were then washed with elution buffer containing increasing concentrations of NaCl: 0.25, 0.5, 0.75, 1, 2M. For each step columns were washed with ten bed volumes, the first 1 ml (Figure 3C) and last 1 ml were kept (Figure S5C). Eluted fractions were concentrated using Nanosep 3KDa (Pall) according to the manufacturer's instructions. Salt concentration was then equalized with ss-DBB. Concentrated protein fractions were recovered in Laemmli buffer and subjected to western blot with the appropriate antibodies (Table S1).

## Supporting Information

Figure S1 (**A**) Schematic representation of MEIOB protein, *Meiob* gene and construction of *Meiob* mutant allele. Grey exons, non coding sequences; black exons, coding sequences (see Materials and Methods section). F and R respectively forward and reverse primers used for RT-qPCR. (**B**) The full length murine *Meiob* transcript was amplified by RT-PCR using specific primers encompassing the ATG and STOP codons. After migration in 1% agarose gel, a single band corresponding to the predicted size (1703 bp) was observed. M, molecular weight marker. (**C**) *Meiob* and *Rfp* mRNA expression in adult *Meiob* ^+/+^, ^+/−^ and ^−/−^ testes. Mean±SEM; n = 2. ND, not detected.
(TIF)

Figure S2 *Meiob* is expressed in gonads. (**A**) *Meiob* mRNA expression was measured by RT-qPCR in various mouse fetal tissues at 13.5 dpc. O, ovary; M, mesonephros; St, stomach; Li, liver; K, kidney; H, heart; B, brain; Lu, lung; Pl, placenta; T, testis. (**B**) *Meiob* mRNA expression was measured by RT-qPCR in different adult mouse organs. T, testis; Li, liver; Sp, spleen; K, kidney; O, ovary; Pa, pancreas; Lu, lung; U, uterus. (**C**) *Meiob* and *Ddx4* mRNA expression were measured using RT-qPCR in 13.5 dpc ovaries of wild type mice (wt) and mice homozygous for *Kit/W* allele (*w/w*) that are devoid of germ cells. Mean±SEM; n = 3. β-*actin* mRNA was used as the endogenous reporter. Data are expressed as a percentage of the maximum mRNA expression.
(TIF)

Figure S3 Hoechst 33342 and propidium iodide (PI) fluorescence profiles of cells from dissociated wild type adult testis acquired by FACS (see Materials and Methods section). Cells were sorted according to the indicated red gates to define “early 4n”, “late 4n” and “2n” populations.
(TIF)

Figure S4 MEIOB localization in chromosome spreads of oocytes at leptotene, zygotene and pachytene stages. Representative chromosome spreads stained for SYCP3 (synaptonemal axial element) and MEIOB protein from 15.5 dpc wild type oocytes. SYCP3 staining was used to visualize the chromosome axes.
(TIF)

Figure S5 (**A**) Subcellular localization of tagged-MEIOB expressed in HEK-293 cells using an anti-Flag antibody. MEIOB protein was observed in both the nucleus and the cytoplasm of the transfected cells. Scale bars, 25 µm. (**B**) Hek-293 cells expressing tagged-MEIOB protein extract was applied to beads coupled with biotine or biotinylated single strand (ss) or double strand (ds) DNA of different lengths (30 mer and 60 mer). Retained proteins were subjected to western blot hybridized with anti-β-ACTIN (green) and anti-c-MYC antibodies (red). Bands intensity quantifications are relative to pull down input protein extract. n = 4 ; Mean±SEM, ***<0.0001; **<0.001 (paired Student's t-test). (**C**) Controls of elution for ssDNA cellulose affinity chromatography presented in figure 3C. Last fractions of each NaCl elution buffer were subjected to western blot (see Materials and Methods section). At the end of each wash, no tagged-MEIOB had been pulled away from the single strand DNA matrix.
(TIF)

Figure S6 Quantification of co-localizations of MEIOB and RPA2 (**A**), DMC1 (**B**) or RAD51 (**C**) in chromosome spreads of wild type leptotene, zygotene and early pachytene spermatocytes from adult testes. For each stage, foci stained for only one protein or for both were counted per cell. The percentage of foci stained for a single or both proteins was then determined. Mean±SEM ; 3 to 13 cells analyzed per stage,
(TIF)

Figure S7 MEIOB and RPA2 (**A**) or ATR (**B**) were detected in chromosome spreads of wild type zygotene and pachytene and *Dmc1* ^−/−^ pachytene-like spermatocytes from adult testes. RPA2/ATR and MEIOB colocalized in *Dmc1* ^−/−^. In *Dmc1* ^−/−^, homologous recombination is arrested prior to strand invasion and the robust MEIOB staining indicates the presence of MEIOB on hyper-resected DNA from the DSB.
(TIF)

Figure S8 (**A**) Testis to body weight ratio in *Meiob* ^+/+^, ^+/−^ and ^−/−^ adult mice. *Meiob* ^−/−^ testis is 3.8 times smaller than *Meiob* ^+/+^ or ^+/−^ testis. Gonads analyzed: n = 8, ***p<0.0001 (paired Student's t-test). (**B**) Section of *Meiob* ^+/+^ and ^−/−^ adult epididymis. *Meiob* ^+/+^ epididymis filled with spermatozoa in contrast to *Meiob* ^−/−^ epididymis with no spermatozoon. Scale bar, 20 µm. (**C**) Seminal vesicle weight of *Meiob* ^+/+^, ^+/−^ and ^−/−^ adult mice. No modification of seminal vesicle weight was observed suggesting no overt alteration of the androgen levels in *Meiob* ^−/−^ adult male mice. Mean±SEM; n = 3.
(TIF)

Figure S9 (**A**) Number of oocytes per *Meiob* ^+/+^, ^+/−^ and ^−/−^ ovaries at 13.5 and 18.5 dpc, at 1, 3 and 10 dpp, and in adult mouse ovaries. The number of oocytes was similar from 13.5 dpc to 1 dpp in mice of different genotypes. At 3 dpp the oocyte number drastically decreased in *Meiob* ^−/−^ ovaries. From 10 dpp and onwards no oocyte was observed in *Meiob* ^−/−^ ovaries. Mean±SEM; Total number of mice analyzed per genotype and per age: at least n = 3. (**B**) Partition of follicle stages in *Meiob* ^+/+^ and ^+/−^ adult mouse ovaries. No change was observed in heterozygous ovaries when compared to wild type. Total number of mice analyzed per genotype: n = 5.
(TIF)

Figure S10 γH2AX and SYCP3 staining in chromosome spreads from *Meiob* ^+/+^ and *Meiob* ^−/−^ in 1 dpp oocytes respectively at pachytene and diplotene and at pachtene-like stages of meiosis prophase I.
(TIF)

Figure S11 RAD51 and DMC1 foci intensities. The intensities of RAD51 and DMC1 foci were measured in wild type and *Meiob^−/−^* spermatocytes at leptotene, early-zygotene, mid-zygotene and late-zygotene/pachytene-like stages. (**A**) Mean of foci intensity per cell and expressed in arbitrary units (AU). In *Meiob^−/−^* the mean intensity of foci tended to decrease in the course of zygotene stage in comparison with *Meiob^+/+^*. (**B**) For each cell intensity were categorized in three groups: Low intensity: <500; Medium intensity: [500–1000[; High intensity: ≥1000 AU. Cells analyzed: 3 to 11 per stage and per genotype. *, p<0.05 and **, p<0.01 (Mann Whitney test, unpaired and nonparametric).
(TIF)

Figure S12 SYCP3 and RAD51 were detected in *Dmc1* ^−/−^ spermatocytes. RAD51 foci persisted in pachytene-like spermatocytes, even those with a high degree of pairing.
(TIF)

Table S1 List of primary and secondary antibodies used in this article for western blot (WB), immunohistochemistry (IHC) and immunofluorescence (IF). P, Polyclonal antibody; M, Monoclonal antibody.
(TIF)

Table S2 Sequences of DNA primers used in this article for genotyping and RT-qPCR.
(TIF)
