# Supplementary figures and images for: MEIOB Targets Single-Strand DNA and Is Necessary for Meiotic Recombination
Source: PLoS Genet. 2013 Sep 19;9(9):e1003784. doi: 10.1371/journal.pgen.1003784 (PMC3778009; doi:10.1371/journal.pgen.1003784)

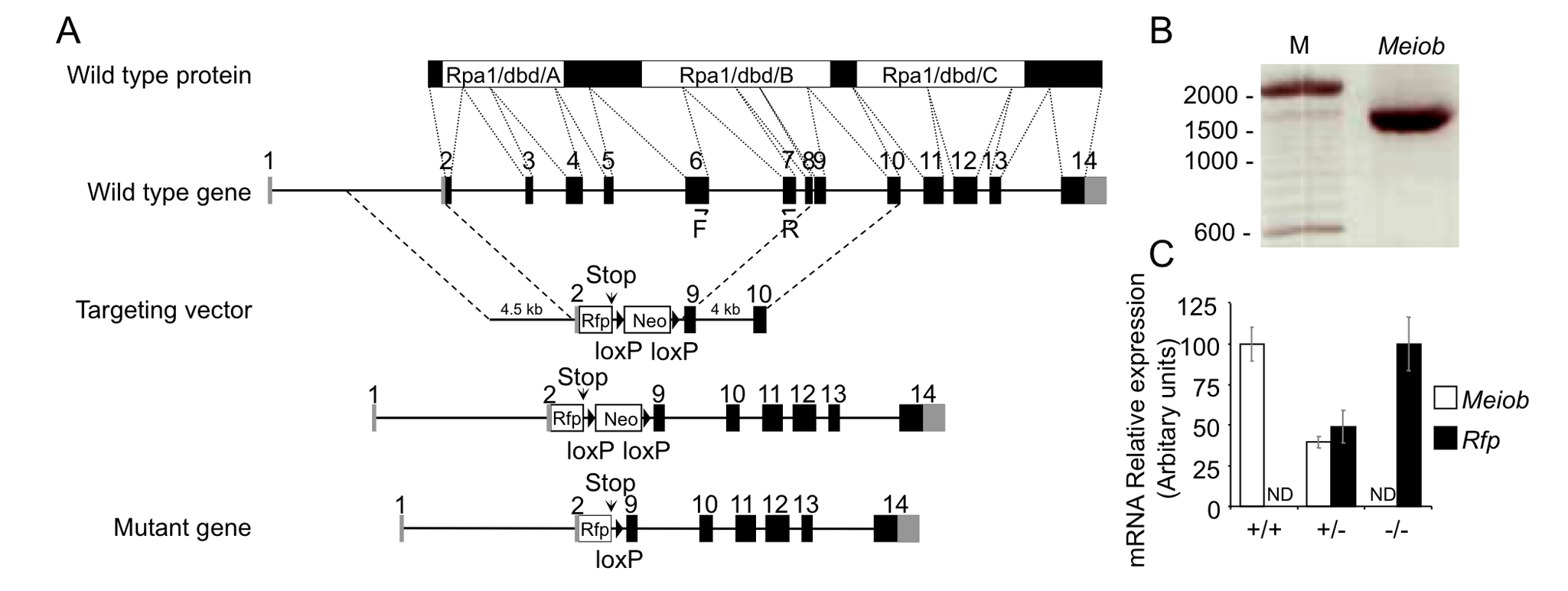

Supplement: Figure S1 — (A) Schematic representation of MEIOB protein, Meiob gene and construction of Meiob mutant allele. Grey exons, non coding sequences; black exons, coding sequences (see Materials and Methods section). F and R respectively forward and reverse primers used for RT-qPCR. (B) The full length murine Meiob transcript was amplified by RT-PCR using specific primers encompassing the ATG and STOP codons. After migration in 1% agarose gel, a single band corresponding to the predicted size (1703 bp) was observed. M, molecular weight marker. (C) Meiob and Rfp mRNA expression in adult Meiob +/+, +/− and −/− testes. Mean±SEM; n = 2. ND, not detected. (TIF) [file pgen.1003784.s001.tif]

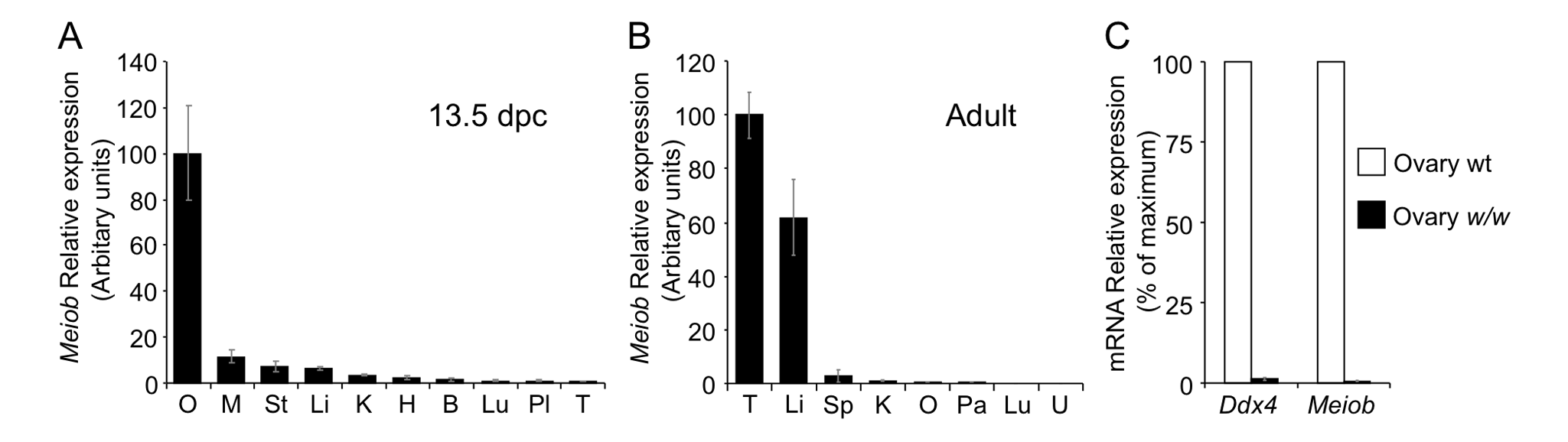

Supplement: Figure S2 — Meiob is expressed in gonads. (A) Meiob mRNA expression was measured by RT-qPCR in various mouse fetal tissues at 13.5 dpc. O, ovary; M, mesonephros; St, stomach; Li, liver; K, kidney; H, heart; B, brain; Lu, lung; Pl, placenta; T, testis. (B) Meiob mRNA expression was measured by RT-qPCR in different adult mouse organs. T, testis; Li, liver; Sp, spleen; K, kidney; O, ovary; Pa, pancreas; Lu, lung; U, uterus. (C) Meiob and Ddx4 mRNA expression were measured using RT-qPCR in 13.5 dpc ovaries of wild type mice (wt) and mice homozygous for Kit/W allele (w/w) that are devoid of germ cells. Mean±SEM; n = 3. β-actin mRNA was used as the endogenous reporter. Data are expressed as a percentage of the maximum mRNA expression. (TIF) [file pgen.1003784.s002.tif]

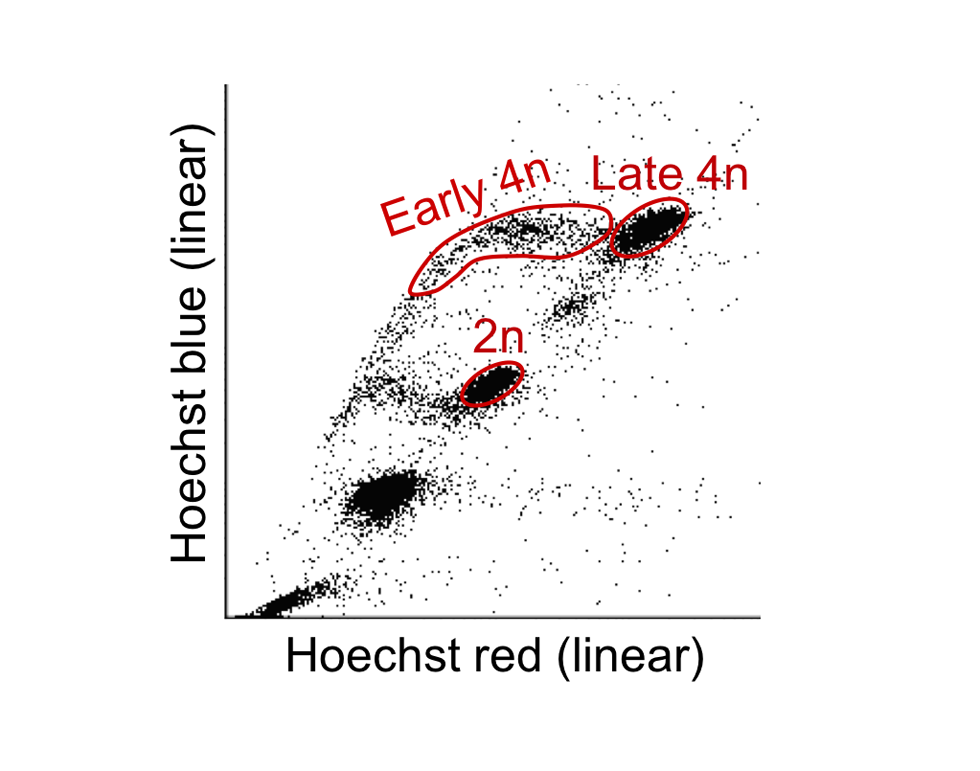

Supplement: Figure S3 — Hoechst 33342 and propidium iodide (PI) fluorescence profiles of cells from dissociated wild type adult testis acquired by FACS (see Materials and Methods section). Cells were sorted according to the indicated red gates to define “early 4n”, “late 4n” and “2n” populations. (TIF) [file pgen.1003784.s003.tif]

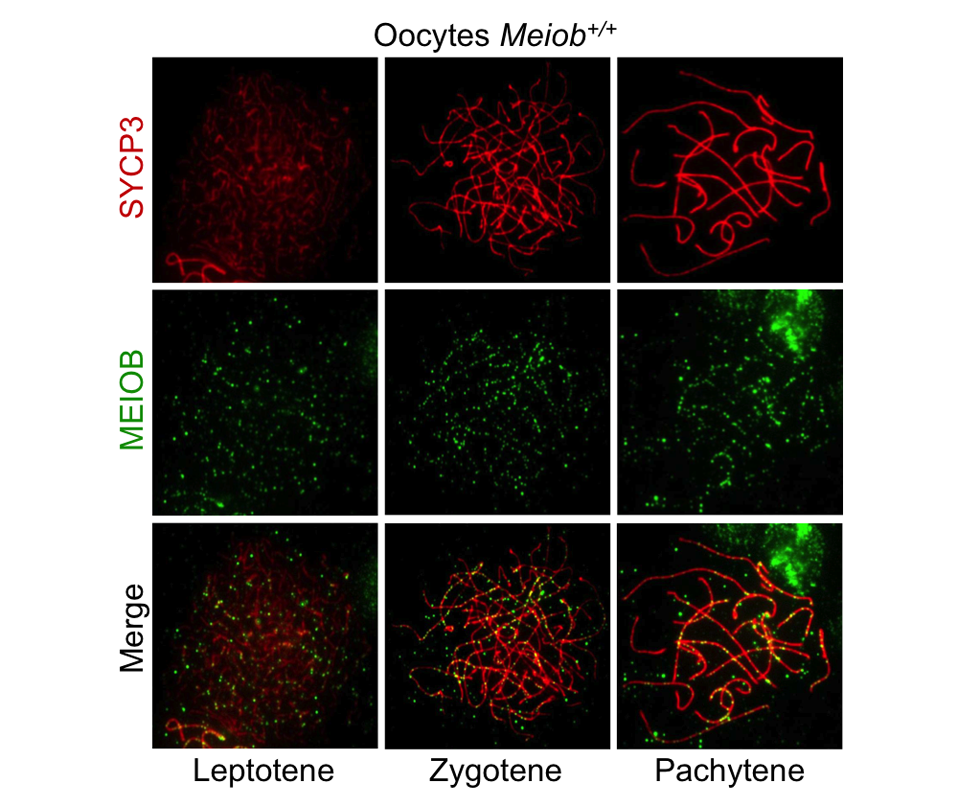

Supplement: Figure S4 — MEIOB localization in chromosome spreads of oocytes at leptotene, zygotene and pachytene stages. Representative chromosome spreads stained for SYCP3 (synaptonemal axial element) and MEIOB protein from 15.5 dpc wild type oocytes. SYCP3 staining was used to visualize the chromosome axes. (TIF) [file pgen.1003784.s004.tif]

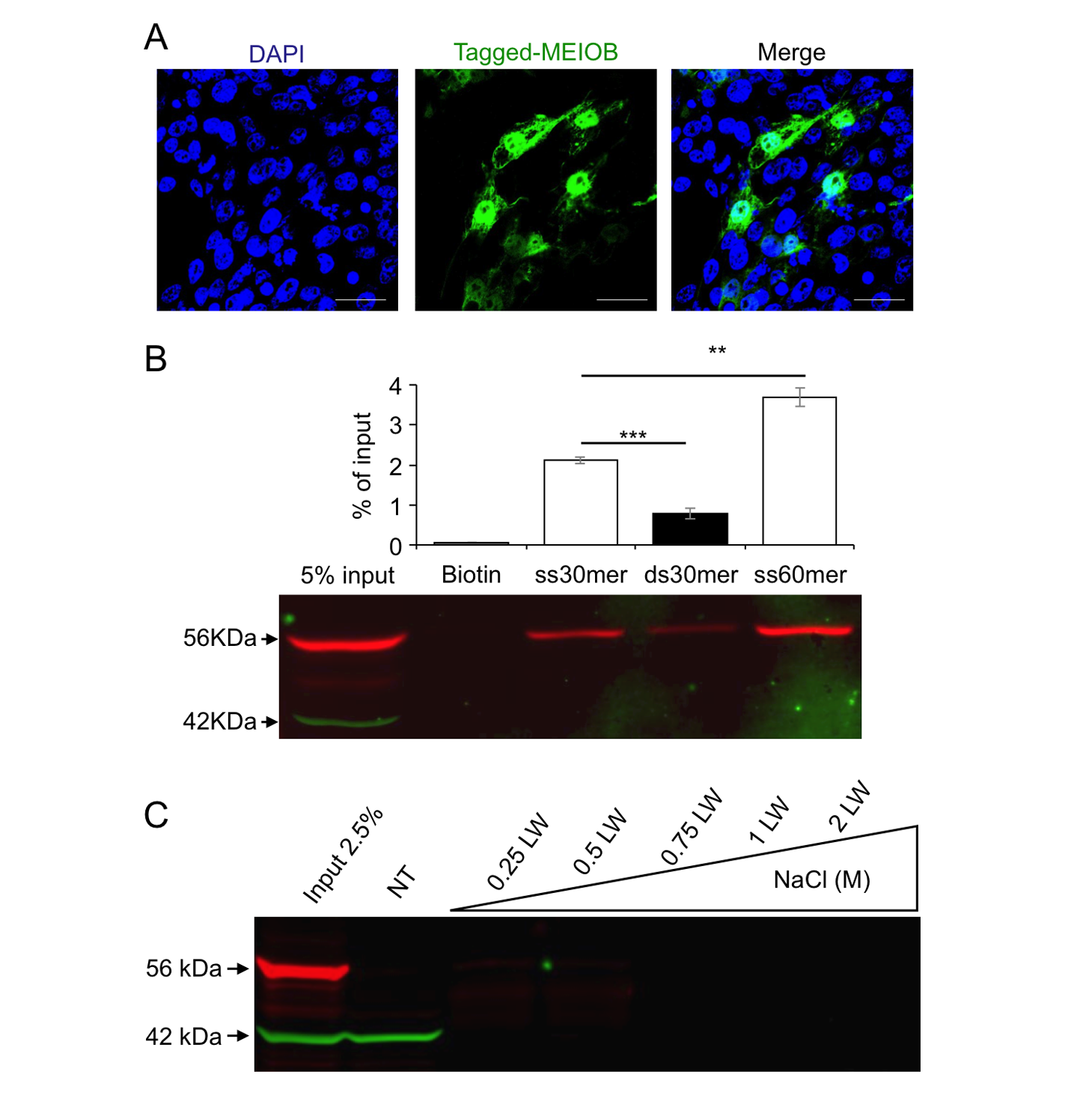

Supplement: Figure S5 — (A) Subcellular localization of tagged-MEIOB expressed in HEK-293 cells using an anti-Flag antibody. MEIOB protein was observed in both the nucleus and the cytoplasm of the transfected cells. Scale bars, 25 µm. (B) Hek-293 cells expressing tagged-MEIOB protein extract was applied to beads coupled with biotine or biotinylated single strand (ss) or double strand (ds) DNA of different lengths (30 mer and 60 mer). Retained proteins were subjected to western blot hybridized with anti-β-ACTIN (green) and anti-c-MYC antibodies (red). Bands intensity quantifications are relative to pull down input protein extract. n = 4 ; Mean±SEM, ***<0.0001; **<0.001 (paired Student's t-test). (C) Controls of elution for ssDNA cellulose affinity chromatography presented in figure 3C. Last fractions of each NaCl elution buffer were subjected to western blot (see Materials and Methods section). At the end of each wash, no tagged-MEIOB had been pulled away from the single strand DNA matrix. (TIF) [file pgen.1003784.s005.tif]

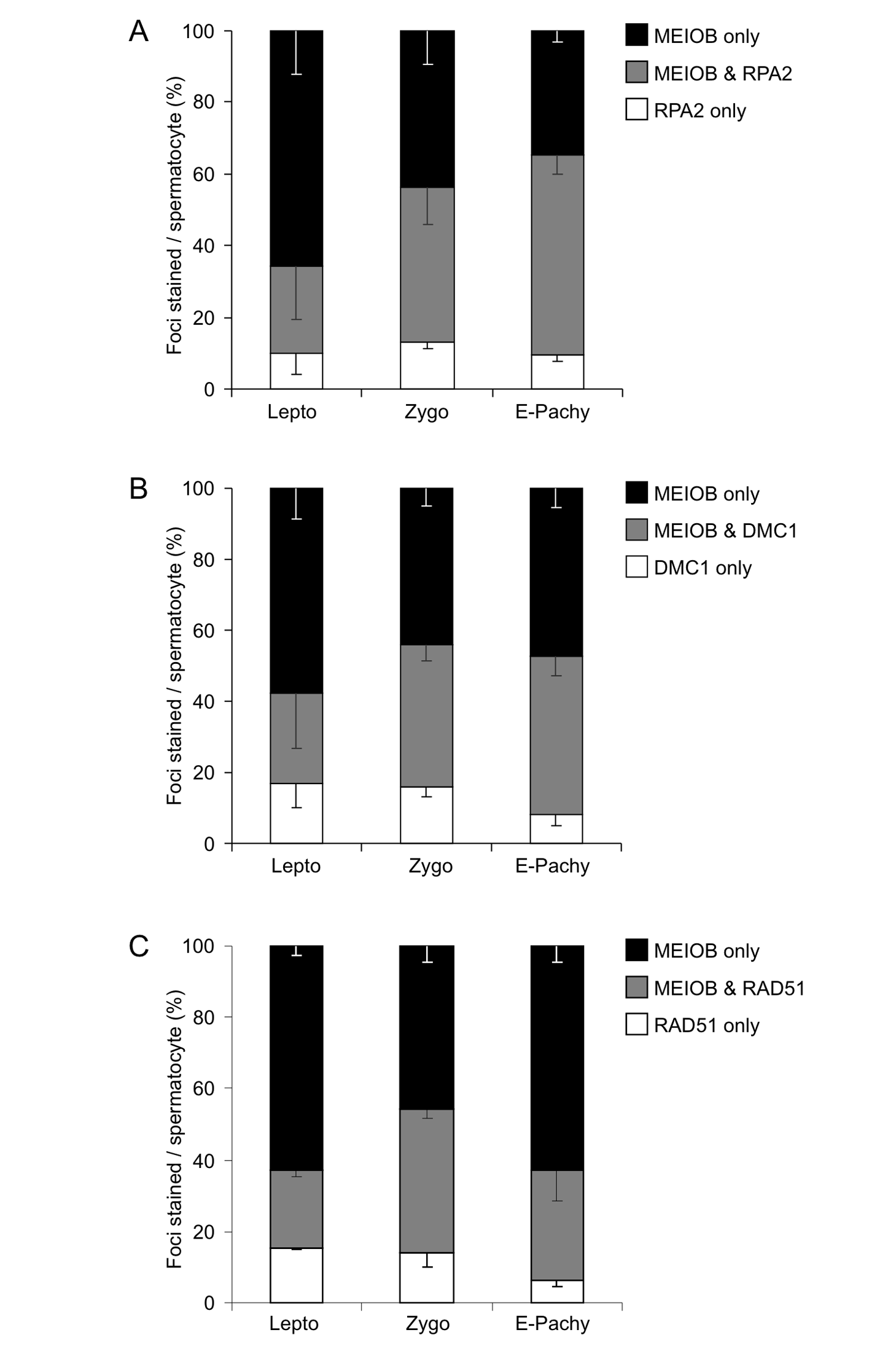

Supplement: Figure S6 — Quantification of co-localizations of MEIOB and RPA2 (A), DMC1 (B) or RAD51 (C) in chromosome spreads of wild type leptotene, zygotene and early pachytene spermatocytes from adult testes. For each stage, foci stained for only one protein or for both were counted per cell. The percentage of foci stained for a single or both proteins was then determined. Mean±SEM ; 3 to 13 cells analyzed per stage, (TIF) [file pgen.1003784.s006.tif]

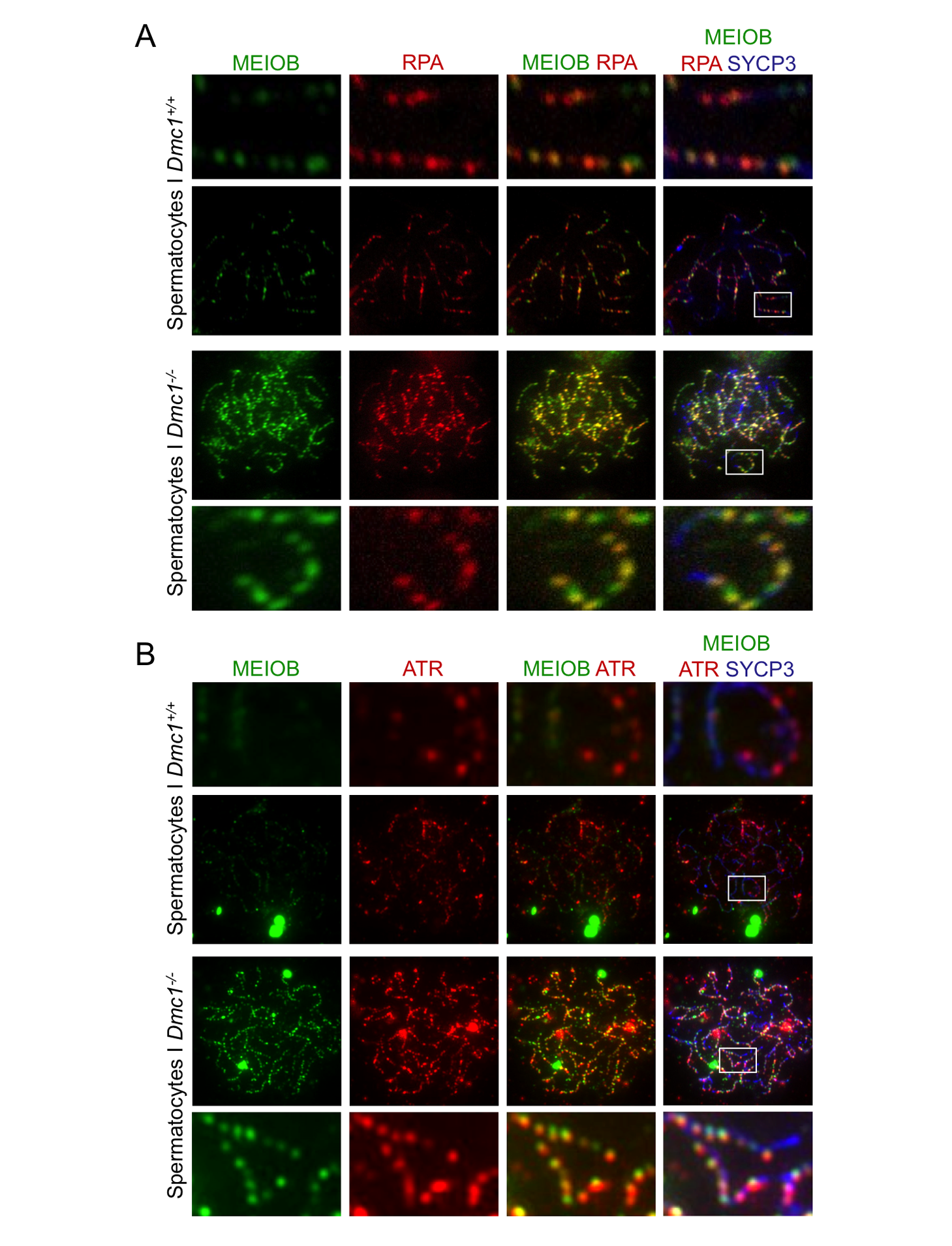

Supplement: Figure S7 — MEIOB and RPA2 (A) or ATR (B) were detected in chromosome spreads of wild type zygotene and pachytene and Dmc1 −/− pachytene-like spermatocytes from adult testes. RPA2/ATR and MEIOB colocalized in Dmc1 −/−. In Dmc1 −/−, homologous recombination is arrested prior to strand invasion and the robust MEIOB staining indicates the presence of MEIOB on hyper-resected DNA from the DSB. (TIF) [file pgen.1003784.s007.tif]

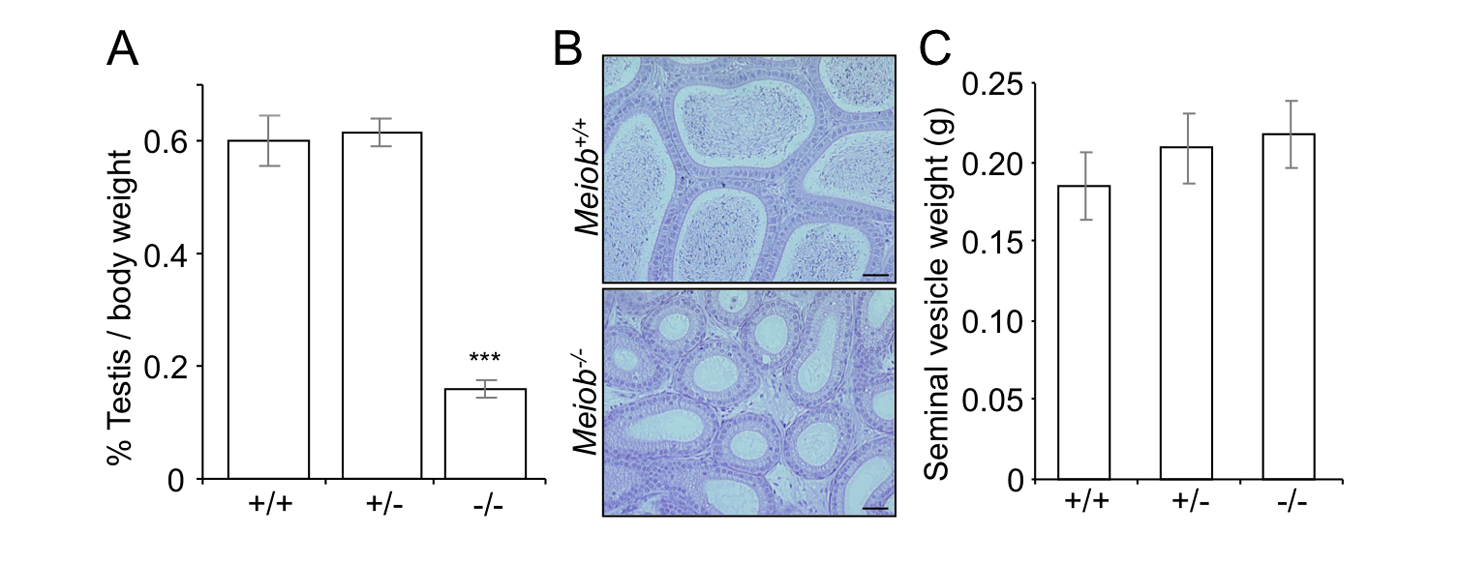

Supplement: Figure S8 — (A) Testis to body weight ratio in Meiob +/+, +/− and −/− adult mice. Meiob −/− testis is 3.8 times smaller than Meiob +/+ or +/− testis. Gonads analyzed: n = 8, ***p<0.0001 (paired Student's t-test). (B) Section of Meiob +/+ and −/− adult epididymis. Meiob +/+ epididymis filled with spermatozoa in contrast to Meiob −/− epididymis with no spermatozoon. Scale bar, 20 µm. (C) Seminal vesicle weight of Meiob +/+, +/− and −/− adult mice. No modification of seminal vesicle weight was observed suggesting no overt alteration of the androgen levels in Meiob −/− adult male mice. Mean±SEM; n = 3. (TIF) [file pgen.1003784.s008.tif]

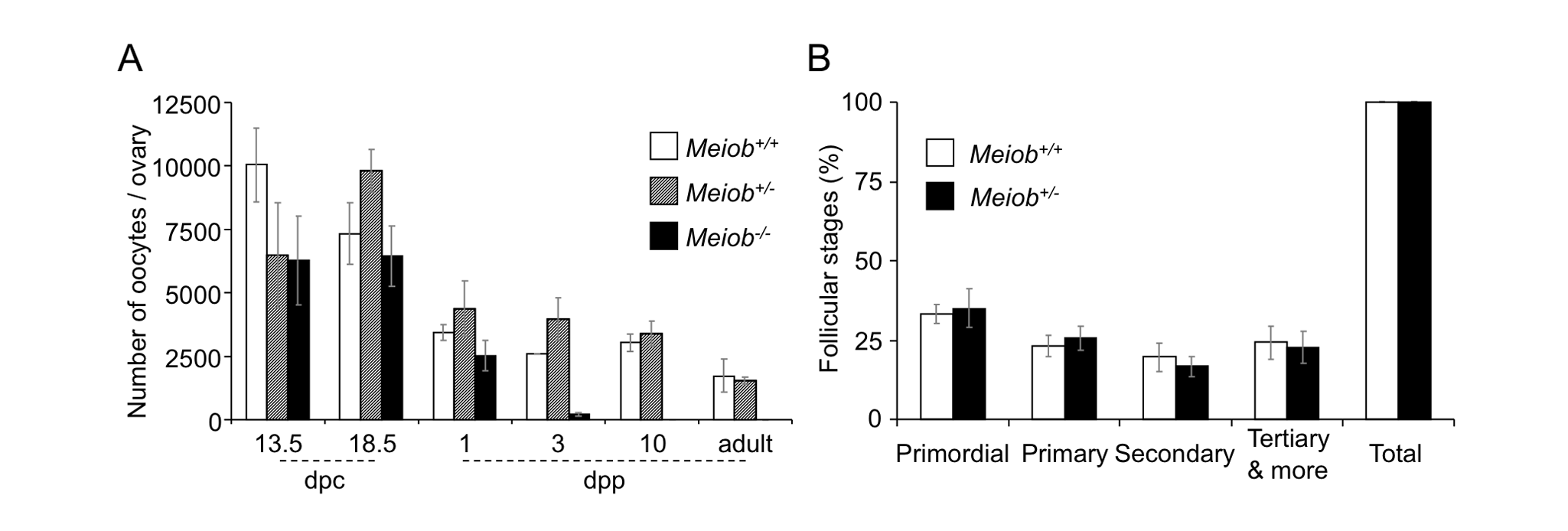

Supplement: Figure S9 — (A) Number of oocytes per Meiob +/+, +/− and −/− ovaries at 13.5 and 18.5 dpc, at 1, 3 and 10 dpp, and in adult mouse ovaries. The number of oocytes was similar from 13.5 dpc to 1 dpp in mice of different genotypes. At 3 dpp the oocyte number drastically decreased in Meiob −/− ovaries. From 10 dpp and onwards no oocyte was observed in Meiob −/− ovaries. Mean±SEM; Total number of mice analyzed per genotype and per age: at least n = 3. (B) Partition of follicle stages in Meiob +/+ and +/− adult mouse ovaries. No change was observed in heterozygous ovaries when compared to wild type. Total number of mice analyzed per genotype: n = 5. (TIF) [file pgen.1003784.s009.tif]

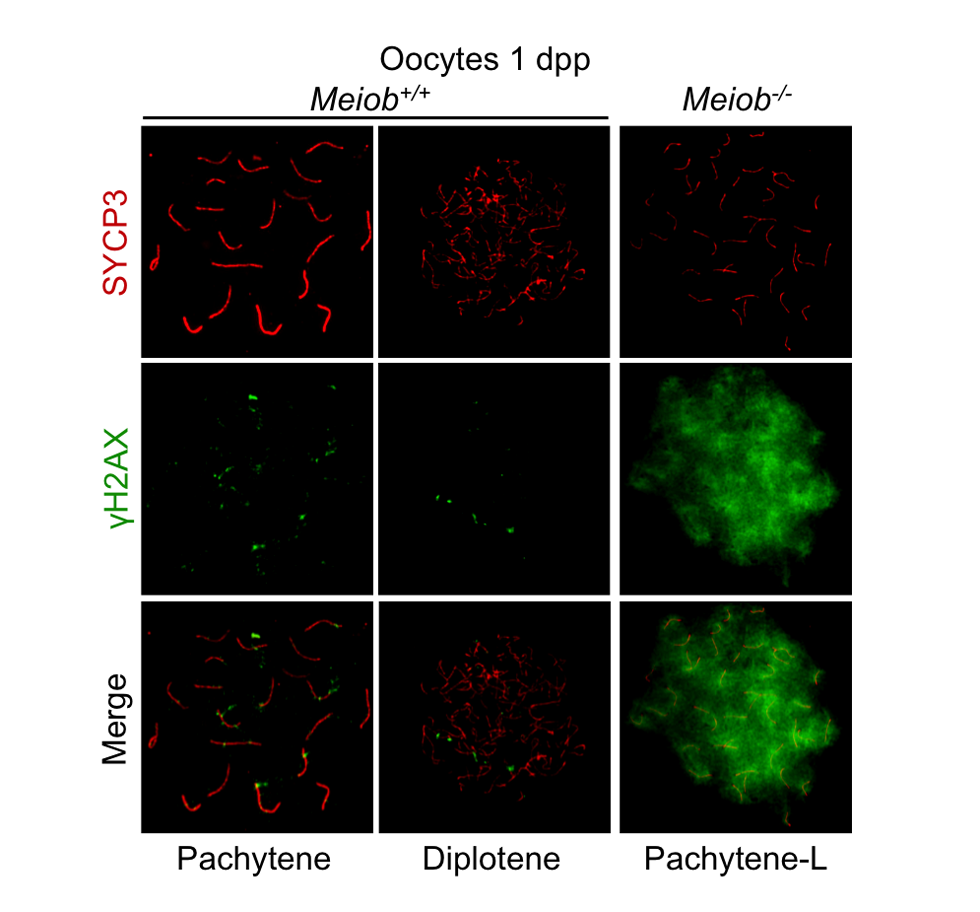

Supplement: Figure S10 — γH2AX and SYCP3 staining in chromosome spreads from Meiob +/+ and Meiob −/− in 1 dpp oocytes respectively at pachytene and diplotene and at pachtene-like stages of meiosis prophase I. (TIF) [file pgen.1003784.s010.tif]

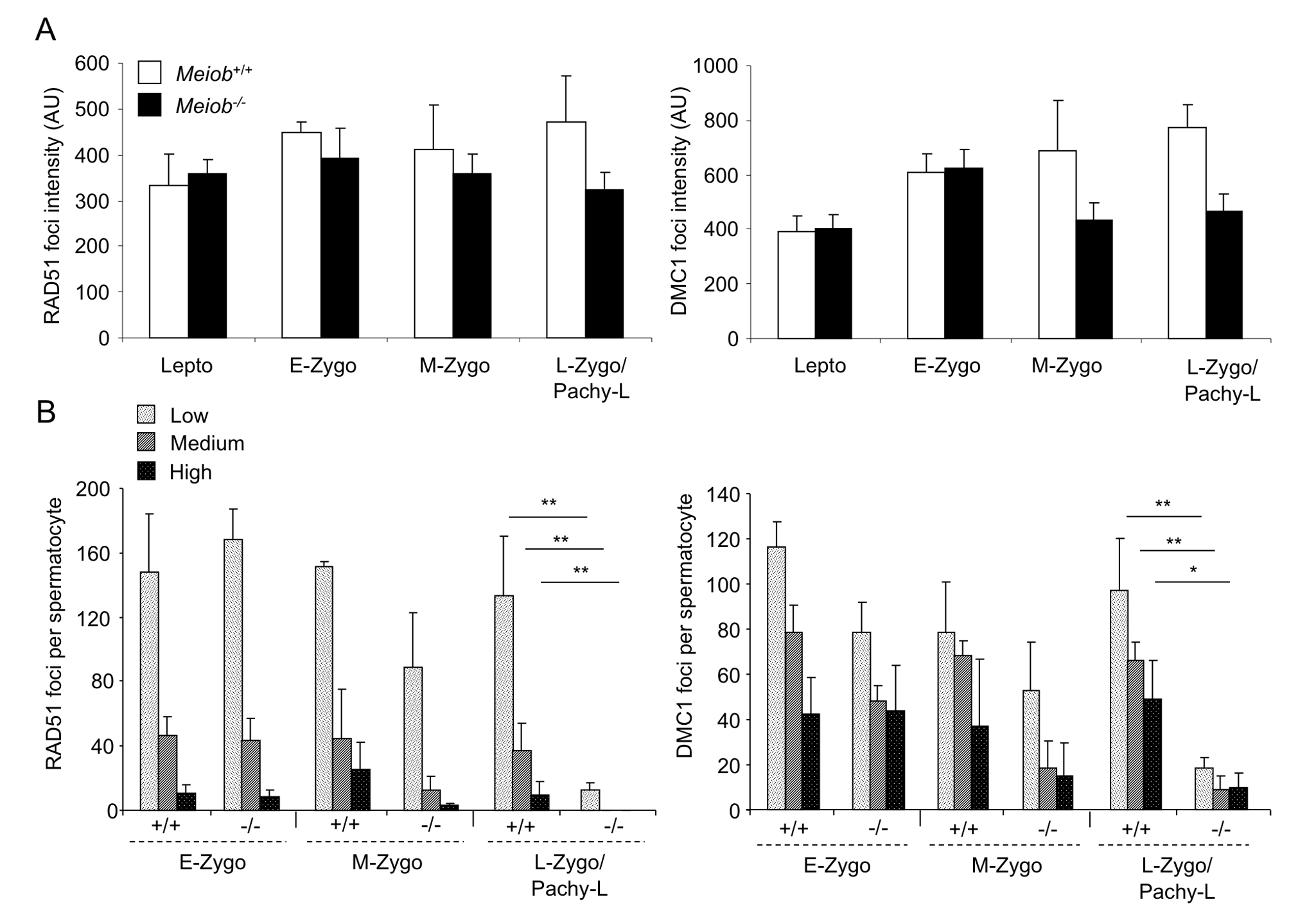

Supplement: Figure S11 — RAD51 and DMC1 foci intensities. The intensities of RAD51 and DMC1 foci were measured in wild type and Meiob−/− spermatocytes at leptotene, early-zygotene, mid-zygotene and late-zygotene/pachytene-like stages. (A) Mean of foci intensity per cell and expressed in arbitrary units (AU). In Meiob−/− the mean intensity of foci tended to decrease in the course of zygotene stage in comparison with Meiob+/+. (B) For each cell intensity were categorized in three groups: Low intensity: <500; Medium intensity: [500–1000[; High intensity: ≥1000 AU. Cells analyzed: 3 to 11 per stage and per genotype. *, p<0.05 and **, p<0.01 (Mann Whitney test, unpaired and nonparametric). (TIF) [file pgen.1003784.s011.tif]

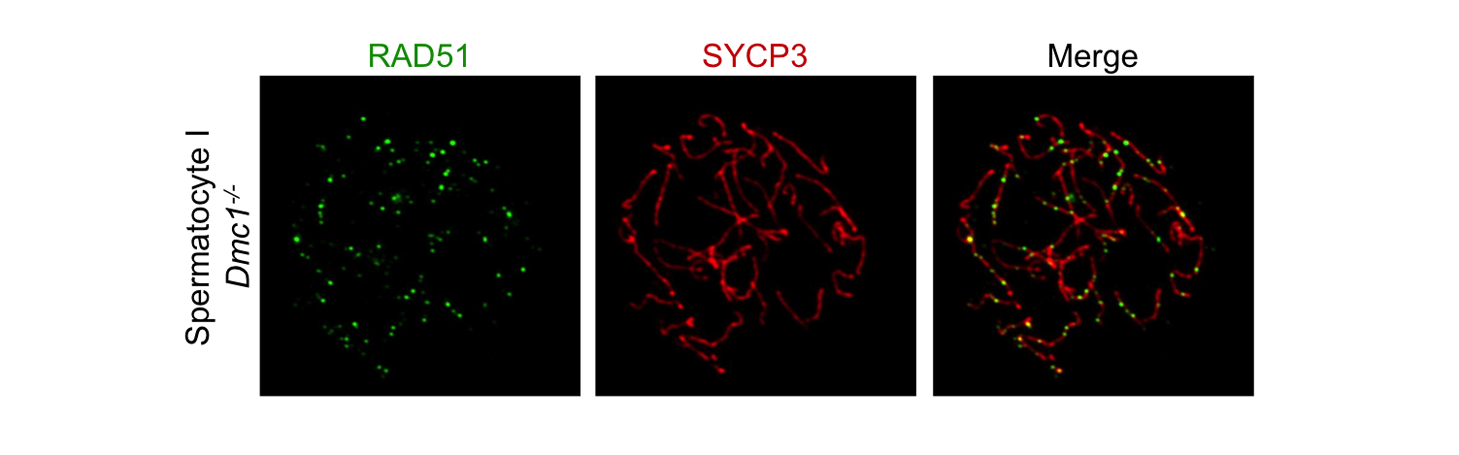

Supplement: Figure S12 — SYCP3 and RAD51 were detected in Dmc1 −/− spermatocytes. RAD51 foci persisted in pachytene-like spermatocytes, even those with a high degree of pairing. (TIF) [file pgen.1003784.s012.tif]

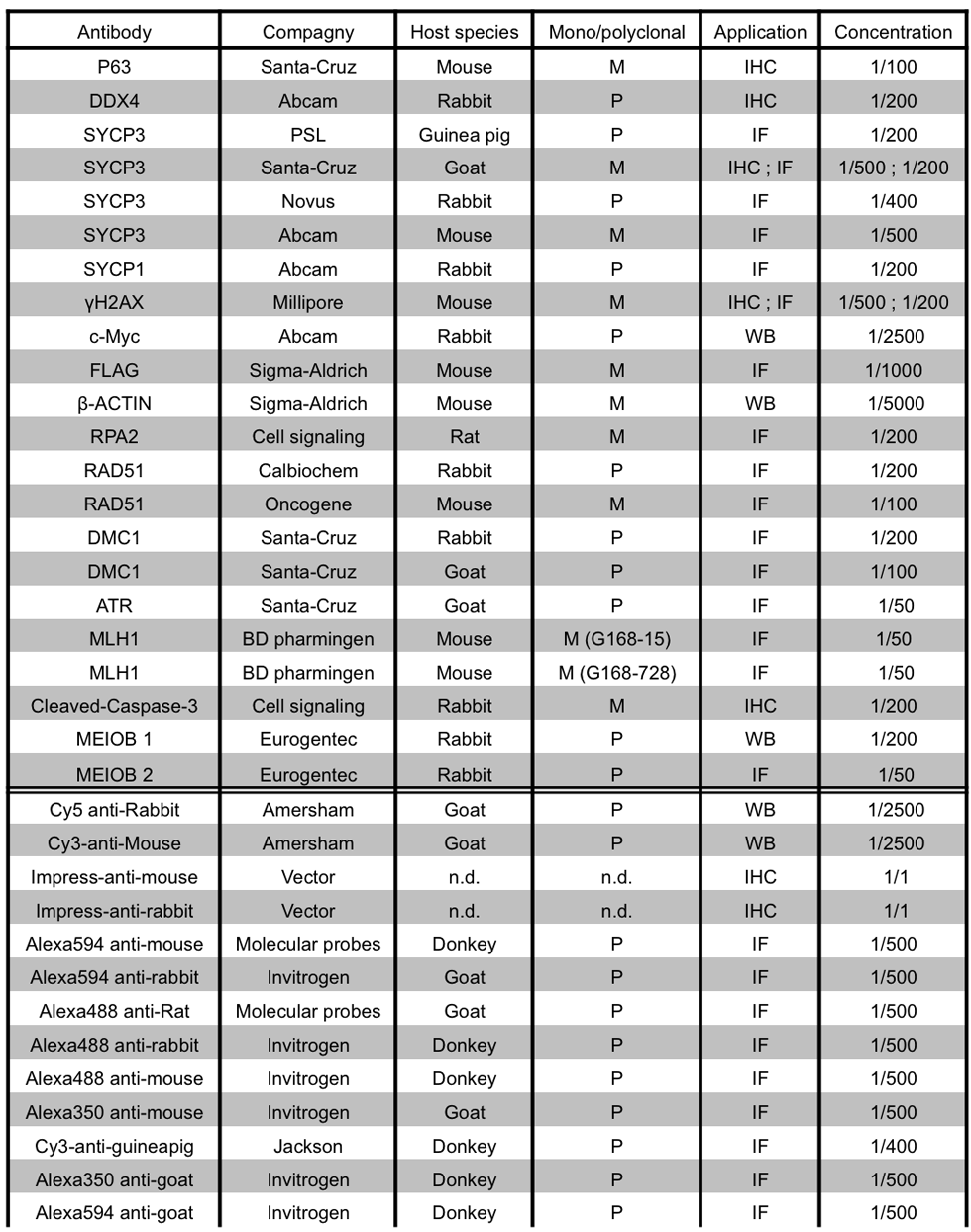

Supplement: Table S1 — List of primary and secondary antibodies used in this article for western blot (WB), immunohistochemistry (IHC) and immunofluorescence (IF). P, Polyclonal antibody; M, Monoclonal antibody. (TIF) [file pgen.1003784.s013.tif]

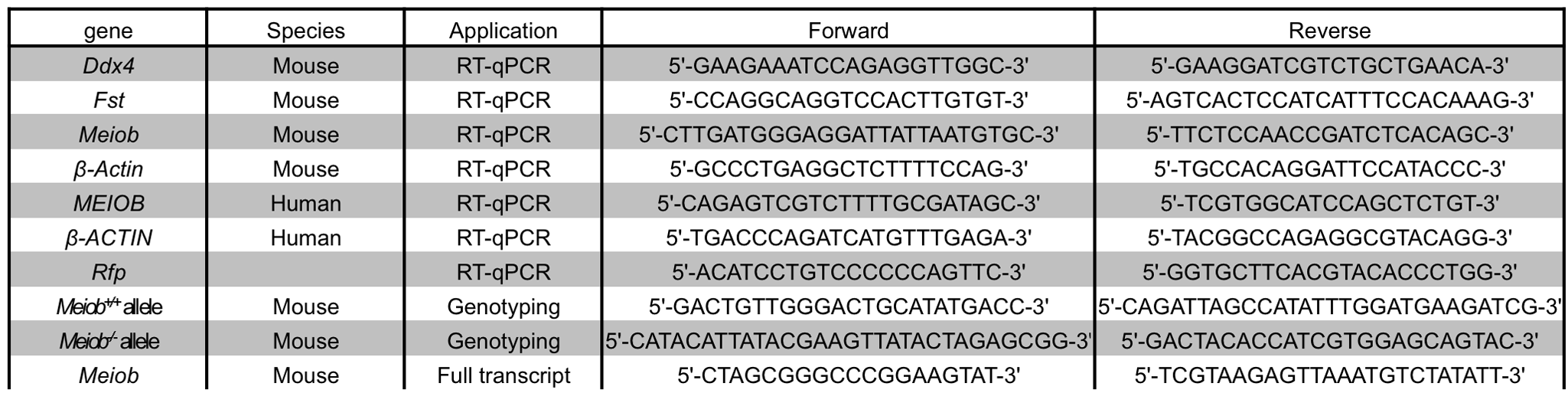

Supplement: Table S2 — Sequences of DNA primers used in this article for genotyping and RT-qPCR. (TIF) [file pgen.1003784.s014.tif]
